# Comparative analyses of new donor-π-acceptor ferrocenyl-chalcones containing fluoro and methoxy-fluoro acceptor units as synthesized dyes for organic solar cell material

**DOI:** 10.1371/journal.pone.0241113

**Published:** 2020-11-04

**Authors:** Ainizatul Husna Anizaim, Dian Alwani Zainuri, Muhamad Fikri Zaini, Ibrahim Abdul Razak, Hazri Bakhtiar, Suhana Arshad

**Affiliations:** 1 X-ray Crystallography Unit, School of Physics, Universiti Sains Malaysia, USM, Gelugor, Penang, Malaysia; 2 Laser Center, Ibnu Sina Institute for Scientific and Industrial Research (ISI-SIR), Universiti Teknologi Malaysia, Johor Bahru, Johor, Malaysia; 3 Department of Physics, Faculty of Sciences, Universiti Teknologi Malaysia, Johor Bahru, Johor, Malaysia; University of New South Wales, AUSTRALIA

## Abstract

Two organometallic compounds known as (*E*)-1-ferrocenyl-(3-fluorophenyl)prop-2-en-1-one (**Fc1**) and (*E*)-1-ferrocenyl-(3-fluoro-4-methoxyphenyl)prop-2-en-1-one (**Fc2**) are designed and synthesized for application in dye-sensitized solar cell (DSSC) based on a donor-π-acceptor (D-π-A) architecture. By strategically introducing a methoxy group into the acceptor side of the compound, **Fc2** which has adopted a D-π-A-AD structure are compared with the basic D-π-A structure of **Fc1**. Both compounds were characterized by utilizing the IR, NMR and UV-Vis methods. Target compounds were further investigated by X-ray analysis and studied computationally using Density Functional Theory (DFT) and Time-Dependent DFT (TD-DFT) approaches to explore their potential performances in DSSCs. An additional methoxy group has been proven in enhancing intramolecular charge transfer (ICT) by improving the planarity of **Fc2** backbone. This good electronic communication leads to higher HOMO energy level, larger dipole moment and better short-circuit current density (J_sc_) values. Eventually, the presence of methoxy group in **Fc2** has improved the conversion efficiency as in comparison to **Fc1** under the same conditions.

## Introduction

Throughout the years, global warming and climate change have been the subject of a great debate around the world which drives to a “game changing” climate agreement poses to humanity. The attention in finding new organic and organometallic materials for various application including photovoltaic performance of dye-sensitized solar cells (DSSCs) have aroused great interest for the energy conversion [1]. DSSC as the third generation of solar cell offer as a promising candidate for an alternative form of renewable solar energy owing to their distinctive aspects of being flexible and cost-effective throughout the entire year conditions [2–5]. To date, the most cell efficiencies in DSSCs are still lower than the established silicon-based and thin-film solar cells. However, according to Bose and co-workers, the performance of the DSSC module highest efficiency discussed was 11.2% for Di-tetrabutylammonium *cis*-bis(isothiocyanato)bis(2,2′-bipyridyl-4,4′-dicarboxylato)ruthenium(II), N719 dye indicating that it is comparable to that Si module [6]. Generally, DSSC involves of a thin layer of fluorine-doped tin oxide (FTO) of a transparent electrode coated with a mesoporous film of nanocrystalline particles of TiO_2_, dye sensitizer, an electrolyte containing a suitable redox couple such as iodide and tri-iodide ions, and a counter electrode [7–9]. The cell represents in a sandwich-like architecture. The dye acts as photosensitizer which plays a crucial role of a DSSC device in absorbing light for the solar energy conversion into electrical energy [9].

**Fig 1:**
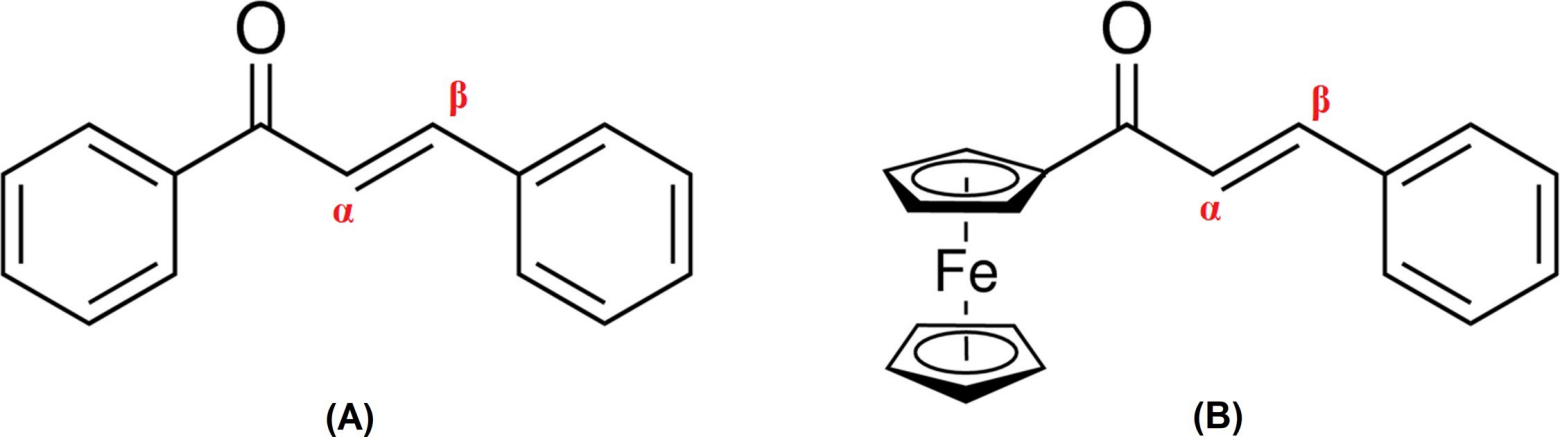
General structure. (A) Chalcone. (B) Ferrocenyl chalcone.

Chalcones are belonging to aromatic ketones family, bearing two aromatic groups associated by α,β-unsaturated system [10] (**Fig 1A**). This chalcone also known as the π-conjugated organic compound signifies appealing attentions for various application in advanced functional materials [11, 12]. The photoconversion efficiencies of DSSCs fabricated using organic dye molecules extremely depending on the molecular structures. Typically, most of the reported structures consist of D-π-A configuration, in which the intramolecular charge transfer (ICT) occurs from the end-capped electron donor (D) to electron acceptor (A) through the π-system [13].

A sandwich compound of dicyclopentadienyl iron, Fe(C_5_H_5_)_2_ possess interesting redox properties as the iron core of Fe is capable to exist in both Fe^2+^ or Fe^3+^ which allowed extensive beneficial applications in energy production, electronic devices and microbiological research [14–19]. Ferrocenyl chalcone (**Fig 1B**) is a very electron rich compound exhibits a good electronic communication of ICT between the donor and acceptor parts. In DSSCs, ferrocene derivatives are considered as good photosensitive dyes for manufacturing of photoanodes as their redox properties enhance a better efficiency in photovoltaic performance [19]. However, less studies were reported regarding on their properties as sensitizer in solar cell application.

Efforts are taken in searching of promising photosensitizer to improve the DSSC efficiency, such as extending the π-conjugation length of the compound (D-π-π-A architecture) and proposing an additional donor (AD) to the main donor (AD-D-π-A architecture) [14]. However, due to some limitations, organic compound with D-π-π-A structure is less preferable than AD-D-π-A structure as it tends to induce the π-π aggregation [20]. A mass of researches has been focused on the design and synthesis of new AD-D-π-A building blocks [20, 21]. For instance, Zhou and co-workers investigated a series of metal-free organic sensitizers using a basic D-π-A architecture with a triphenylamine moiety serves as the electron donor, tetrathienoacene as the π-bridge unit, and a cyanoacrylic acid as the electron acceptor [21]. Expanding the π-conjugation length by inserting an additional thiophene as the AD reduced the respective band gaps and increased the absorption wavelength. Based on the reported findings, the authors proved that an additional donor to the donor or acceptor sides for D-π-A structure will eventually give almost the same results [20, 21].

Although many have suggested additional donor for example methoxy group into the main donor induces a bathochromic shift in the absorption spectrum which improves photovoltaic performances [22, 23], some additional donor can also be introduced into the acceptor part. An addition of donor substituent to the main donor reduced core planarity and hindered charge injection, leading to a poorer electron injection efficiency for DSSC performance. However, an addition of donor to the acceptor side slightly enhanced the planarity of the compound and its orientation on TiO_2_, resulting an improvement of electron injection efficiency and dye loading density on the TiO_2_ surface [21]. Besides, Fu and co-workers also investigated that the introduction of electron-donating group into the D-π-A backbone leads the HOMO level to be shifted up, in which consequently improve the charge transport properties and photovoltaic performance [24].

In the past few years, quantum chemical calculations have gained a great attention as a reliable alternative to interpret experimental data and at the same time offer some predictable properties of new materials [24]. One of the methods that dominates this field is called density functional theory (DFT), owes its popularity due to its often-good electronic correlation and can applied to the organic macromolecular systems at an acceptable cost [25, 26].

With these views in mind, we herein report two new structural ferrocenyl chalcones of **Fc1** and **Fc2** that were successfully synthesized. By strategically introducing a methoxy group as the additional donor (AD) into the acceptor side of the ferrocenyl chalcone, the electronic structures of the molecule can be readily tuned. **Fc1** represented as the non-methoxy substituted ferrocenyl chalcone having a D-π-A structure, while **Fc2** showed a methoxy substituted ferrocenyl chalcone with a new modification of D-π-A-AD structure. The dyes have ferrocene derivatives as a main donor (D), enone moiety as a π-bridge (π), fluorophenyl ring as an acceptor (A), and methoxy group as an additional electron donor (AD). We expect that the introduction of methoxy group as an additional donor unit in the linear D-π-A organic dye can effectively enhance the open-circuit voltage (V_oc_) of the DSSC with good efficiency. As aforementioned above, an additional methoxy group is also expected to enhance the planarity of the framework which then improve the charge transfer within the compound, lead to bathochromic shift of the absorption spectrum and increase the HOMO level of the molecule. The molecular structure of the ferrocenyl chalcones were studied by X-ray structural analysis, Infrared (IR) vibrational spectra and UV-Visible. Additionally, computational DFT investigation with B3LYP/6-311G++ (d,p) basis set was carried out to gain further insight into their effects and rationalize the results. We believe the performance of methoxy substitution ferrocenyl chalcone (**Fc2**) in light harvesting properties will be higher than non-methoxy substitution ferrocenyl chalcone (**Fc1**).

## Materials and methods

### Synthesis and crystal growth

**Scheme 1:**
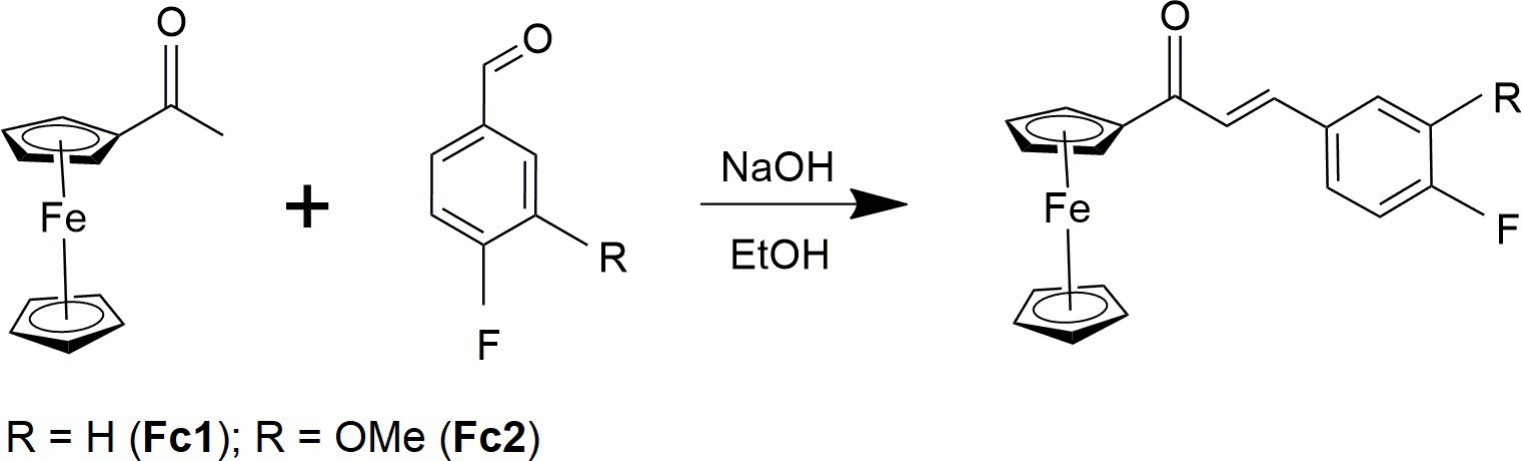
The formation of ferrocenyl chalcones of Fc1 and Fc2.

The designed **Fc1** and **Fc2** were prepared by Claisen Schmidt condensation method as outlined in **Scheme 1**[27]. All solid and liquid reagents (Merck, Aldrich) handled in preparations were used without any further purification. Both compounds were synthesized in the same fashion. A mixture of 1-acetylferrocene (3 mmol) and corresponding aldehydes (3 mmol), 3-fluorobenzaldehyde (**Fc1**) and 3-fluoro-4-methoxybenzaldehyde (**Fc2**) were used and dissolved in ethanol (10 mL), respectively. A catalytic amount of diluted NaOH was added to the solution dropwise with vigorous stirring. In both cases, the reaction mixture turned into dark reddish-brown. The reaction mixture was stirred overnight at room temperature. The reaction progress was monitored by TLC until completion to determine if the starting materials have been consumed and a new product has been formed. The resultant precipitate is collected by vacuum filtration, washed successively with distilled water and purified by recrystallization from acetone to yield the corresponding ferrocenyl chalcone of **Fc1** (86.2%) and **Fc2** (77.2%).

### Spectroscopic analyses

The infrared spectra (FT-IR) were recorded by the ATR method with a PerkinElmer GX Frontier Spectrophotometer in the range of 600–4000 cm^-1^. The UV-Vis spectra were obtained at ambient temperature on a UV-Visible Spectrophotometer Model Cary 5000 UV-Vis-NIR in the wavelength region of 200–800 nm at a concentration of 10^−4^ mol L^-1^ in 1 cm cuvette. The ^1^H and ^13^C NMR measurements were performed at room temperature in DMSO-*d*_6_ using a Bruker 500 and 125 MHz Avance III spectrometer, respectively. The chemical shifts (*δ*) are reported in parts per million (ppm) downfield from tetramethyl silane (TMS) internal reference.

(*E*)-1-ferrocenyl-(3-fluorophenyl)prop-2-en-1-one (**Fc1**), C_19_H_15_FFeO. Deep-red solid: yield 86.2% (1.11 g); IR (ATR-FTIR, cm^-1^): 3149 (Cp C–H), 3087 (Ar–H), 1650 (C = O), 1593 (C = C), 1273 (C–F). ^1^H-NMR (500.13 MHz; DMSO-*d*_6_; ppm): δ 7.63–7.50 (m, 4H, Ph), 7.27 (1H, s, H-*α*), 6.55 (d, 1H, H-*β*), 5.06 (2H, s, Ph-Fe), 4.67 (2H, s, Ph-Fe), 4.21 (5H, s, Ph-Fe). ^13^C NMR (125.76 MHz, DMSO-*d*_6_, ppm): δ 192.41 (C = O), 138.83 (C-*β*), 132.65 (C-*α*), 81.01, 73.36, 70.30, 70.20 (C-Fe).

(*E*)-1-ferrocenyl-(3-fluoro-4-methoxyphenyl)prop-2-en-1-one (**Fc2**), C_20_H_17_FFeO_2_. Deep-red solid: yield 77.2% (0.937 g); IR (ATR-FTIR, cm^-1^): 3064 (Cp C–H), 2840 (Ar–H), 1652 (C = O), 1591 (C = C), 1275 (C–F). ^1^H-NMR (500.13 MHz; DMSO-*d*_6_; ppm): δ 7.88–7.58 (m, 3H, phenyl), 7.35 (1H, s, H-*α*), 6.53 (d, 1H, H-*β*), 5.04 (2H, s, Ph-Fe), 4.63 (2H, s, Ph-Fe), 4.19 (5H, s, Ph-Fe), 3.87 (3H, s, CH_3_). ^13^C NMR (125.76 MHz, DMSO-*d*_6_, ppm): δ 192.34 (C = O), 153.10 (C-*β*), 127.18 (C-*α*), 81.23, 73.09, 70.21, 69.51 (C-Fe).

### X-ray crystallography determination

The grown crystals were characterized by single crystal X-ray diffraction (XRD) analysis performed on ApexII Duo CCD area-detector diffractometer using MoKα radiation (λ = 0.71073 Å) are summarized in **(S1 File)**. Data collections were carried out using the APEX2 software [28] proceeded with the cell refinement and data reduction utilizing the SAINT software [28]. The crystal structures were solved by Direct Method using the program SHELXL [29] and was refined by full-matrix least squares technique on *F*^2^ using anisotropic displacement parameters by SHELXL [29]. Absorption correction was applied to the final crystal data using the SADABS software [28]. All geometrical calculations were carried out using the program PLATON [30]. The molecular graphics were drawn using SHELXL [29] and Mercury program [31]. Anisotropic thermal factors were assigned to all non-hydrogen atoms. All H atoms in **Fc1** and **Fc2** were positioned geometrically with the bond lengths of C–H being 0.93–0.98 Å and refined using a riding model with *U*_iso_(H) = 1.2 *U*_eq_ (C) and 1.5 *U*_eq_(C_methyl_).

### Quantum chemical calculations: DFT studies

GAUSSIAN 09 program was utilized [32] for all theoretical calculations of both compounds by taking the initial geometrical parameters obtained from Single Crystal X-ray Diffraction refinement data. The optimization of the molecular geometries leading to energy minima was achieved using the DFT [with Becke’s non-local three parameter exchange and the Lee-Yang-Parr’s correlation functional (B3LYP)] with the 6–311++G(d,p) basis set for C, O, F and H atoms and LANL2DZ functional for Fe, under vacuum. The optimized structural parameters were used to calculate the vibrational wavenumbers. The calculated vibrational frequencies corresponded to potential energy minima wherein no attainable imaginary frequency and the theoretical results were scaled down by a uniform scaling factor of 0.972 (**Fc1**) and 0.971 (**Fc2**) for frequencies less than 1700 cm^-1^ and 0.971 (**Fc1**) and 0.935 (**Fc2**) for higher frequencies. Also, the time dependent density functional theory (TD-DFT) at 6–311++G(d,p) has been employed in HOMO-LUMO energies, oscillator strengths, absorption wavelengths λ_max_. The geometry optimization was performed in gas-phase and employing a polarizable continuum model (PCM) using the integral equation formalism variant (IEFPCM) by considering the solvent environment to determine the effect of the DMSO solvent on the equilibrium molecular geometry within the molecules. Furthermore, the molecular electrostatic potential (MEP), Mulliken and ground state dipole moment have also been calculated with the same level of theory which is B3LYP/6-311++G(d,p) basis set.

### Dye-sensitized solar cell (DSSC) applications

**Fig 2:**
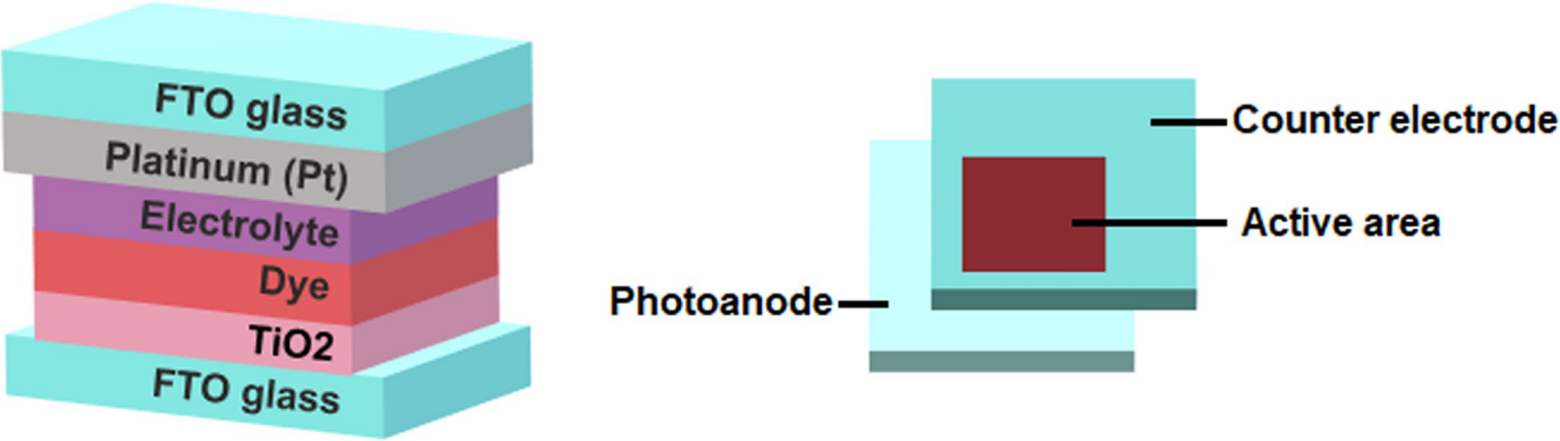
A schematic diagram of the DSSC structure.

A ready-to-use DSSC kit (purchased from Solaronix). A schematic diagram of the basic structure of DSSC is shown in **Fig 2**. Dyes are prepared from a mixture of respective compound **Fc1** and **Fc2** with acetonitrile, respectively. About 6mm x 6mm area each of TiO_2_ coated on FTO glass were sensitized by dipping them in a respective dye solution. The process occurred overnight, then all samples were rinsed with excess acetonitrile to remove the redundant dye and dried in hot-air flow. A frame of 60m sealing film made from Meltonix 1170 60 μm thick sealing film was put between the photoanode and counter electrode. The sandwich-type cell was prepared via melting the sealing film on a hot plate while applying pressure gradually. The presence of predrilled hole in the counter electrode was used to inject an iodide based redox electrolyte (Iodolyte AN-50, Solaronix) by the capillary action using a syringe into the sandwich cell. Then hole was sealed with another piece of sealing film to avoid the electrolyte leakage and dried-out dye. All the cells were tested under the irradiation of AM 1.5 simulated sunlight (CHF-XM-500 W) at intensity of 100 ± 3mW/cm^2^. A source meter (Keithley 2400) was adopted to measure the output voltage and current of the cells. Electrochemical impedance spectroscopy (EIS) was performed on a Interface1000 Potentiostat (Gamry Instruments) in a two-electrode configuration. The photoanode was connected as a working electrode and the platinum (Pt) electrode as a counter electrode. The electron transport properties were investigated using EIS with a 10 mV AC signal in the frequency range of 10 mHz to 1 MHz.

## Results and discussion

### Molecular and optimized structure analysis

**Fig 3:**
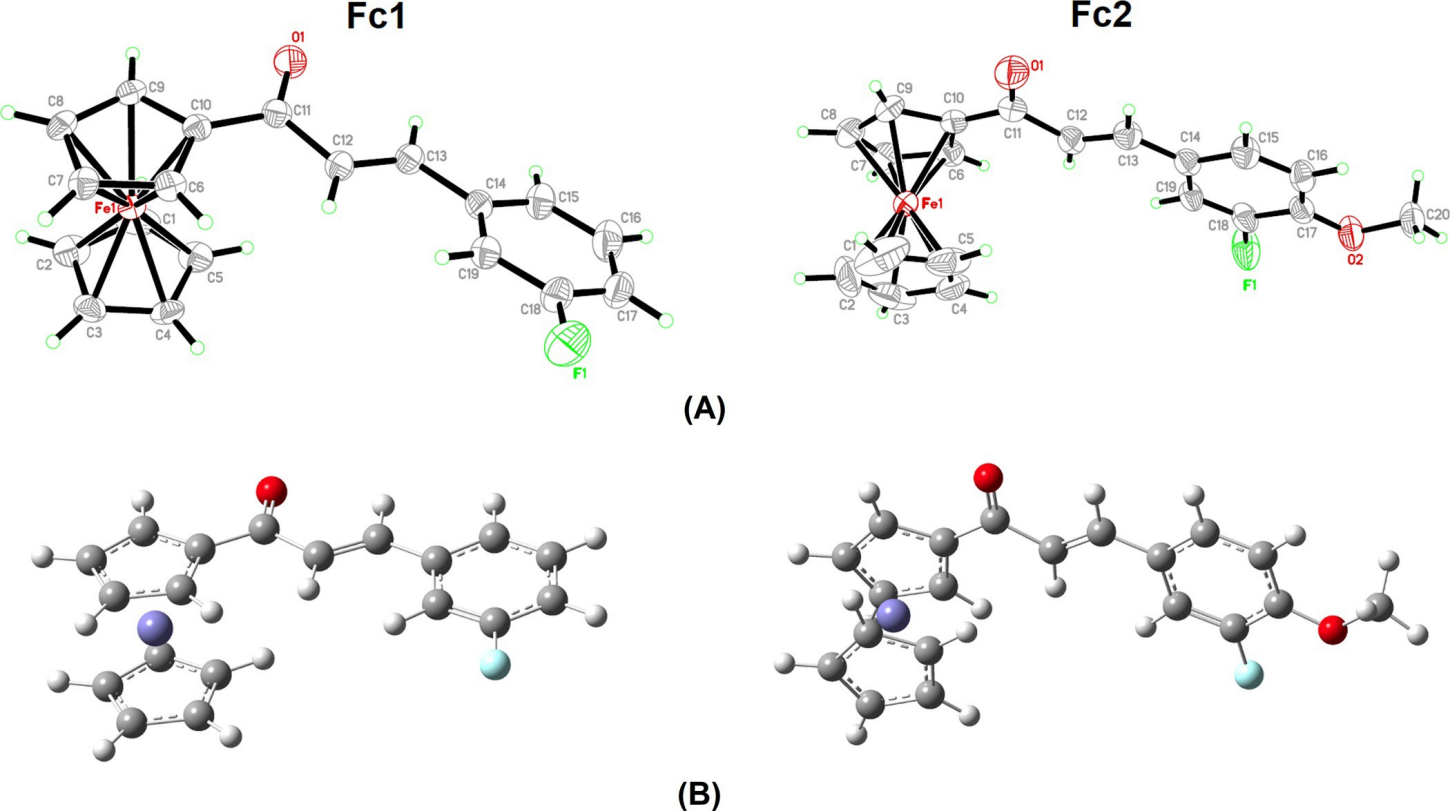
The molecular structure of Fc1 and Fc2. (A) ORTEP diagrams showing 50% displacement ellipsoids (B) Optimized structures using DFT/B3LYP/6-311G++(d,p) basis set level.

Crystallization of the compounds yield the deep red crystals with needle and plate morphology for **Fc1** and **Fc2**, respectively (**S1 Fig in S1 File**). Detailed molecular structures of both compounds were confirmed by X-ray analysis. The ORTEP diagrams and optimized structures of **Fc1** and **Fc2** are shown in **Fig 3**. The experimental bond lengths, bond angles, and dihedral angles as well as the optimized structures obtained by B3LYP methods combining with 6-311G++(d,p) basis set for **Fc1** and **Fc2** are tabulated in **Table 1**. All theoretical data attained show a good agreement with the experimental data.

**Table 1:** Selected experimental and calculated bond lengths and angles.

	Fc1		Fc2	
	Experimental	DFT	Experimental	DFT
Bond Lengths (Å)				
Fe1—C10	2.020 (5)	2.069	2.014 (3)	2.073
Fe1—C1	2.037 (6)	2.078	2.000 (5)	2.081
F1—C18	1.362 (8)	1.356	1.360 (3)	1.348
O1—C11	1.216 (7)	1.225	1.221 (4)	1.225
C1—C2	1.417 (10)	1.427	1.364 (11)	1.427
C2—C3	1.423 (10)	1.426	1.343 (10)	1.426
C3—C4	1.380 (9)	1.426	1.375 (6)	1.426
C4—C5	1.406 (10)	1.426	1.345 (6)	1.426
C1—C5	1.411 (10)	1.425	1.349 (8)	1.425
C6—C10	1.427 (8)	1.440	1.430 (5)	1.440
C6—C7	1.415 (8)	1.420	1.421 (5)	1.421
C7—C8	1.419 (8)	1.427	1.404 (6)	1.428
C8—C9	1.412 (7)	1.418	1.411 (6)	1.419
C9—C10	1.416 (7)	1.434	1.435 (5)	1.433
C10—C11	1.484 (9)	1.480	1.475 (4)	1.480
C11—C12	1.479 (8)	1.487	1.482 (4)	1.482
C12—C13	1.320 (7)	1.344	1.323 (4)	1.342
C13—C14	1.478 (7)	1.463	1.457 (4)	1.456
Fe1—C(Cp1) avg	2.041	2.078	2.007	2.081
Fe1—C(Cp2) avg	2.040	2.078	2.034	2.081
O2—C17			1.355 (4)	1.352
O2—C20			1.421 (4)	1.424
Bond Angles (°)				
C9—C10—C11	123.6 (5)	123.6	123.6 (3)	123.7
C6—C10—C11	127.7 (5)	129.2	128.1 (1)	129.0
O1—C11—C12	120.9 (6)	121.6	121.1 (3)	121.9
O1—C11—C10	121.8 (6)	120.7	121.3 (3)	120.4
C12—C11—C10	117.3 (5)	117.7	117.7 (3)	117.7
C13—C12—C11	122.0 (5)	120.8	122.2 (3)	120.8
C12—C13—C14	126.3 (5)	127.9	128.1 (3)	128.0
C19—C14—C13	122.2 (5)	122.9	122.6 (3)	123.2
C15—C14—C13	119.7 (5)	118.8	120.4 (3)	119.2
C17—C18—F1	118.7 (6)	118.6	116.3 (3)	118.1
F1—C18—C19	117.6 (6)	118.6	119.8 (3)	119.5
C17—O2—C20			117.8 (3)	118.1
Torsion Angles (°)				
C9—C10—C11—C12	-167.9 (5)	-174.4	163.4 (3)	169.0
C6—C10—C11—C12	4.8 (9)	2.7	-9.8 (4)	-7.6
C10—C11—C12—C13	178.1 (5)	-179.0	-176.0 (3)	-179.3
C11—C12—C13—C14	175.2 (5)	-179.7	-179.4 (3)	178.2
C12—C13—C14—C19	-15.1 (8)	-0.3	1.3 (5)	1.0
C12—C13—C14—C15	166.7 (6)	169.6	-179.8 (3)	-178.4
O1—C11—C12—C13	-3.2 (9)	0.9	2.8 (5)	-0.1
O2—C17—C18—F1			1.0 (4)	-0.1

Cp1: C1 – C2 –C3 – C4 – C5; Cp2: C6 – C7 – C8 – C9 – C10.

Both compounds (**Fc1** and **Fc2**) consist of a ferrocene group, an enone group and an aryl halide group (fluorobenzene). In addition, **Fc2** comprises of an additional methoxy group anchoring to the fluorobenzene ring. Bond lengths and angles are almost similar in both compounds and within the expected range [33]. The X-ray structural analysis of **Fc1** and **Fc2** imply that they are crystallized in the orthorhombic crystal system with *Pna*2_1_ [*a* = 20.601(3) Å, *b* = 12.3027(15) Å, *c* = 5.8821(7) Å] and monoclinic crystal system with P2_1_/*n* [*a* = 11.1429(8) Å, *b* = 7.3917(5) Å, *c* = 20.3228(15) Å, *β* = 101.892(3)°] space group, respectively. Both structures (**Fc1** and **Fc2**) comprise of four molecules per unit cell (*Z* = 4). In the present ferrocenyl chalcones, the cyclopentadienyl rings (Cp1 and Cp2) in both rings **Fc1** and **Fc2** adopt a nearly eclipsed geometry. The values of the torsion angles C5—Cp1—Cp2—C10 are 2.94° and 7.18° in **Fc1** and **Fc2**, respectively. Furthermore, the planes of the Cp rings are standing nearly parallel on top of each other, having a dihedral angle of Cp1/Cp2 [C1—C5/C6—C10] 3.297 (3)° and 3.278 (3)° in **Fc1** and **Fc2**, respectively. These corresponding angles which are all below 3.30° showed that the pairs of Cp rings exhibit only a slight mutual sloping between them [34, 35].

The crystal structures of **Fc1** and **Fc2** illustrate that the most stable configuration of (*E*)-isomer lie within the C12 = C13 bond. The enone moiety (O1/C11—C13) of **Fc1** and **Fc2** adopts *s*-cis configuration with respect to O1 = C11 and C12 = C13 bonds. Both compounds **Fc1** and **Fc2** are slightly twisted at the C10—C11 bonds with the C9—C10—C11—C12 torsion angles of 167.9(5)° and 163.4(3)°, respectively. However, the corresponding torsion angles for DFT reveal that the compounds are almost coplanar (**Table 1**), -174.4 (**Fc1**) and 169.0 (**Fc2**). Stem from the fact that the optimization is performed in isolated conditions while the experimental XRD are easily affected by the crystal environment and hydrogen-bonding interactions, thus some angle difference might occur [27].

In both **Fc1** and **Fc2**, the C–C bonds within the Cp rings are found to be consistent. 1.407 Å and 1.355 Å are the average values for this bond within the unsubstituted ring, Cp1 in **Fc1** and **Fc2**, respectively (**Table 1**). The corresponding DFT results give the same average values of 1.426 Å for both compounds. Meanwhile, the interesting feature of slightly higher average values of C–C bonds within another substituted ring, Cp2 are 1.416 Å (**Fc1**) and 1.420 Å (**Fc2**) owing to the displacement of the ring which closed to the enone moiety. Consequently, the DFT results show that both **Fc1** and **Fc2** having a similar average value of C–C bonds in Cp2 which is 1.428 Å. This trend indicates that the delocalization of the Cp ring charge occurred only over the substituent atom, whereby the effect is not transmitted to the unsubstituted free ring. Also, the consistent average values for both compounds in theoretical geometry (DFT) are due to the values are obtained from gas-phase calculation in which the crystal packing forces are eliminated [36]. Based on the Cp2 ring, C6—C10 and C9—C10 are found to be the longest bonds as they are assigned in the vicinity of the carbonyl substituent bonded to C10 atom (**Table 1**). From the previous literature, the maximum deviations of the cyclopentadienyl ring fused to the enone moiety planes are reported shall not exceed 20° [37].

**Fig 4:**
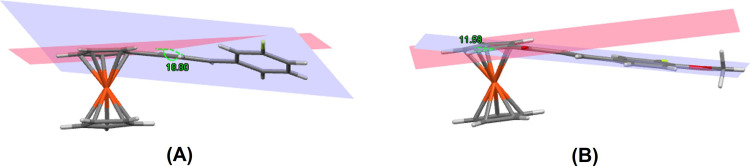
The angle between substituted Cp ring plane and phenyl ring plane. (A) Fc1 (B) Fc2.

The angle between carbonyl moiety, C11—O1 and Cp2 plane are slightly twisted. As take the consideration between the angle of the carbonyl vector and the Cp2 plane itself, the values are found to be 10.44° (**Fc1**) and 13.96° (**Fc2**). On the other hand, the angle of the vector coincides with the C11—O1 and the phenyl fragment is much larger for **Fc1**, 20.24°. Interestingly, the case is different for **Fc2** as the value obtained is significantly lower which is 4.49°. The 3-fluorophenyl substituent of **Fc1** is found to be slightly twisted with the enone moiety at C13–C14 bond with the experimental and theoretical C12—C13—C14—C15 torsion angles being 166.7 (6)° and 169.6°, respectively. Whereas, the 3-fluoro-4-methoxyphenyl substituent of **Fc2** is observed to be almost co-planar with the enone moiety at C13–C14 bond with the C12—C13—C14—C15 torsion angles of -179.8 (3)° experimentally and 178.6° theoretically (DFT). As expected, the presence of heavy substituent of methoxy group anchoring to the phenyl ring of **Fc2** is responsible to the distortion of the molecular planarity in the compound. The insertion of additional donor to the acceptor side can slightly tune the molecular backbone planarity of the structure and the substituent group tends to form an intermolecular C—H···O hydrogen bond which confines the enone moiety and the substituted ring into a near planar conformation [12]. The angle between the plane defined by ring of 3-fluorophenyl group and Cp2 ring bonded to this group in **Fc1** is found to be 18.80°, whereas in **Fc2** the angle between 3-fluoro-4-methoxy group and the substituted Cp2 ring is observed to be 11.58° (**Fig 4**). Tracking down a planar molecule is considered as the most fruitful approach in attaining high intramolecular charge transfer within the conjugated compounds [38].

**Fig 5:**
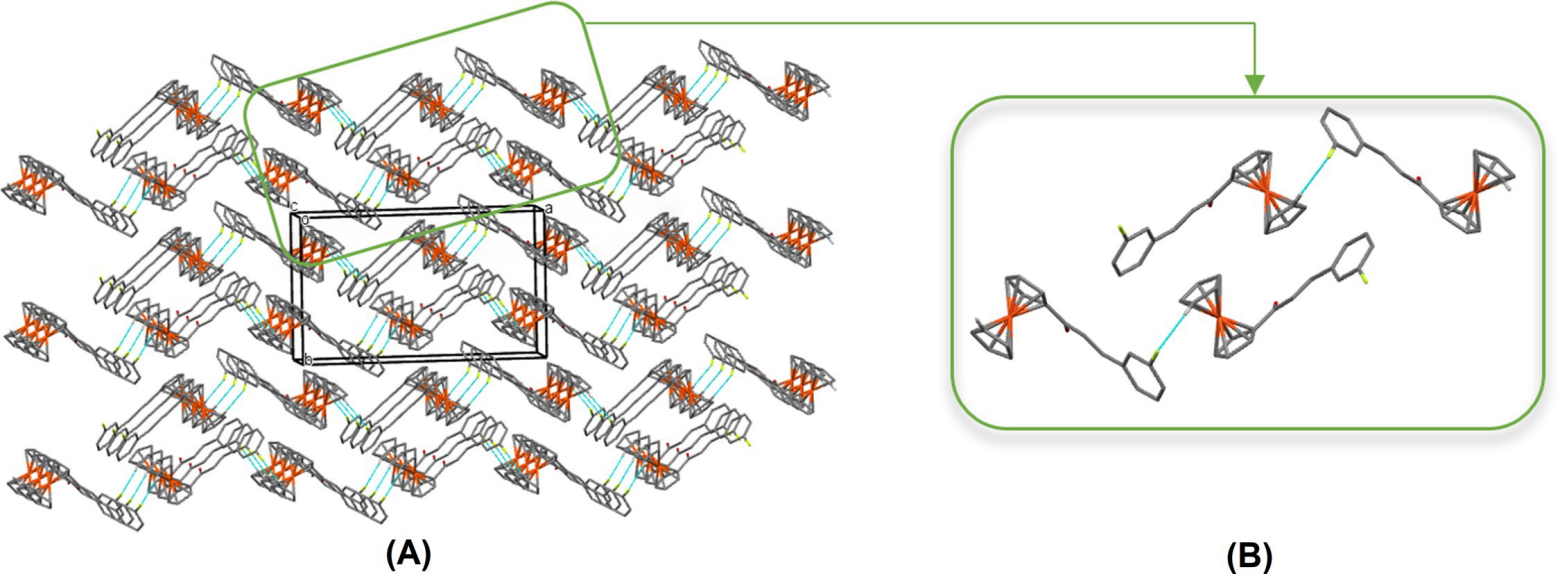
Crystal packing of Fc1. (A) Full view within a unit cell. (B) A partial view along the *a*-axis of the crystal packing. The C—H···F hydrogen bonds are shown as cyan dashed lines.

The formation of weak hydrogen bond is constrained to C—H···O interactions mainly with the existence of carbonyl group in the enone moiety, a group that is identified as one of the best proton acceptors in weak hydrogen bonds [39]. Likewise, the presence of a fluorine atom in the molecules creates the possibility of C—H···F interactions, whereas the presence of aromatic rings can be involved in C—H···π and π—π contacts [40, 41]. This study revealed that there are no significant C—H···O, C—H···π or π—π interactions, but only weak C—H···F interactions are presence in **Fc1** (**Table 2**). The extension of this interaction through the molecular centre of inversion leads to sheets of molecules, which the molecules are arranged into a head to tail fashion propagating along the *a*-axis (**Fig 5**). Also, the molecules are directed parallel along *a*-axis with alternating directions for adjacent rows forming a three-dimensional framework of **Fc1**. Therefore, it appears that the crystal packing in **Fc1** is mainly dominated by intermolecular C3—H3*A*···F1 interaction to stabilize the compound. In the crystal lattices, all the aforementioned hydrogen bonds and intermolecular interactions play a significant role in stabilizing the crystal structures.

**Table 2:** Hydrogen Bond interactions of the compounds.

Bond	Bond length (Å)			Angle
D—H⋯A	D—H	H⋯A	D⋯A	D—H⋯A (°)
**Fc1**				
C3—H3*A*⋯F1^i^	0.93	2.61	3.537	158
**Fc2**				
C19—H19*A*⋯F1^ii^	0.93	2.55	3.396	152
C4—H4*A*⋯F1^ii^	0.93	2.59	3.459	148
C20—H20*A*⋯*Cg*1^iii^	0.93	2.80	3.597	140
C20—H20*C*⋯O1^iv^	0.93	2.64	2.637	157

Symmetry code: ^(i)^ 1/2+*x*, 3/2-*y*, *z*

^(ii)^ 1-*x*, 1-*y*, 1-*z*;

^(iii)^ -1/2+*x*, 3/2-*y*, -1/2+*z*;

^(iv)^ -*x*, 1-*y*, 1-*z*.

*Cg*1 is the centroid of the C1-C5 ring.

**Fig 6:**
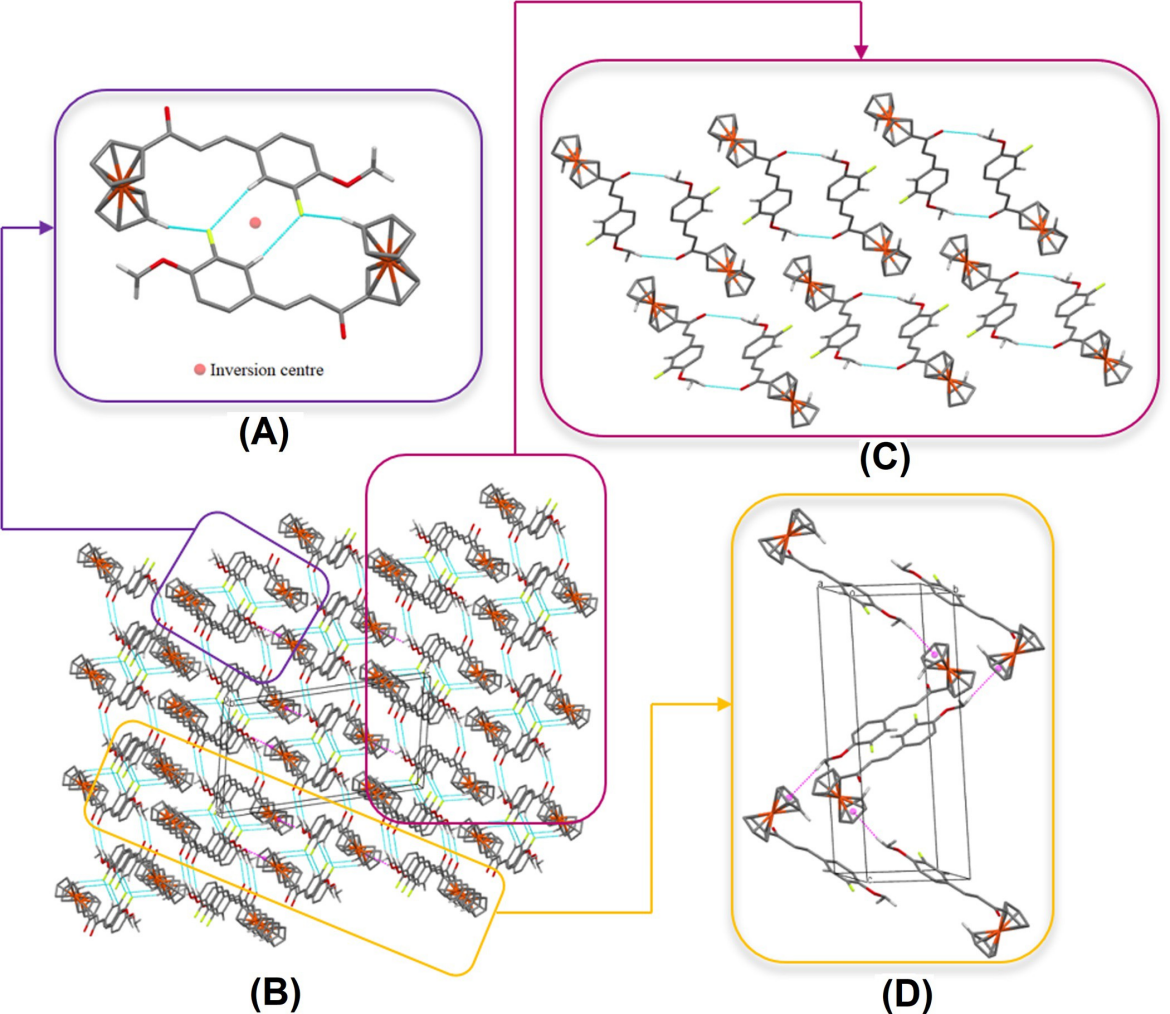
Crystal packing of Fc2. (A) A view of a centrosymmetric dimer with weak intermolecular C19—H19*A*⋯F1 and C4—H4*A*⋯F1 interactions shown as blue dotted lines (B) A full view of crystal packing within a unit cell (C) A partial parts of C20—H20*C*⋯O1 interactions (D) The head-to-tail arrangement of C20—H20*A*⋯*Cg*1.

In the crystal packing of **Fc2**, two adjacent molecules are linked into centrosymmetric dimers by weak C19—H19*A*⋯F1 interactions *via* the inversion centre forming an *R*_2_^2^ (8) ring motif (**Fig 6A and Table 2**). Considering that the distance between atoms C4 and F1 is relatively less than 3.80Å, C4—H4*A*⋯F1 interactions are also involved in further stabilized the adjacent molecules. These dimers are further linked into double-stranded chains by weak C20—H20*C*⋯O1 interactions, the atom O1 of the molecule at (–*x*, 1 –*y*, 1 –*z*) serves as an acceptor for atom C20 of the molecule in the asymmetric unit which generate molecular wires along the [100] axis (**Fig 6B**). The introduction of methoxy substituent caused the formation of new hydrogen bonds, in which the O atom or the H atoms of the methoxy group were directly involved [42]. The chains generate layers of molecules in the *ac-*plane (**Fig 6C**). At the same time, the methoxy group, *via* C20—H20*A* has an edge-on intermolecular C—H⋯π interaction with the centroid of the unsubstituted Cp ring, C1–C5 (*Cg*1) at -1/2 + *x*, 3/2 –*y*, -1/2 + *z*. Also, the molecules are arranged into zig-zag chains by this weak C20—H20*A*⋯*Cg*1 interaction, in which the head-to-tail chains are running down the *ac*-plane (**Fig 6D**). The additional methoxy substituent is not only improved the planarity the compound but also offered a room for more interactions to occur, thus they further stabilize the crystal structure.

### Fourier transform infrared spectroscopy (FTIR) analysis

FTIR analysis helps to determine various kinds of functional groups in molecules by assigning their critical vibrational modes [43]. Normally, the higher wavenumbers are due to the high vibration of stronger bonds and light atoms. In general, the behavior of covalent bonds which act like springs in organic molecules can be run-down mathematically into specific vibrational modes. A selection of essential measured and calculated vibrational frequencies for the study compounds along with corresponding vibrational assignments are given in **Table 3**. The calculated FTIR spectra were carried out by DFT B3LYP/6-311++G(d,p) basis set level with the values obtained are comparable with the experimental values. The calculated harmonic frequencies were scaled by 0.971 (**Fc1**) and 0.935 (**Fc2**) for higher frequencies and 0.972 (**Fc1**) and 0.971 (**Fc2**) for frequencies less than 1700 cm^-1^ [44].

**Table 3:** Assignment of IR.

IR assignments	Fc1			Fc2		
	Unscaled IR frequency (cm^-1^)	Scaled IR frequency (cm^-1^)	FTIR (ATR) (cm^-1^)	Unscaled IR frequency (cm^-1^)	Scaled IR frequency (cm^-1^)	FTIR (ATR) (cm^-1^)
*v*(C = O)	1714.26	1666.26	1650.04	1718.49	1668.65	1651.97
*v*(C = C)	1639.87	1593.95	1593.17	1640.93	1593.34	1591.33
*v*(C–H)	3149.00, 3179.65	3149.00, 3087.44	3149.00, 3087.05	3063.88, 3038.04	3063.88, 2839.66	3063.88, 2839.70
*v*(C–O)	**-**	**-**	**-**	1306.89	1268.99	1275.15
*b*(C–H)	1285.34, 1243.74, 1188.72, 1070.87	1249.35 (Cp), 1208.92, 1155.44, 1040.89 (Cp)	1250.25, 1215.98, 1145.86, 1031.42	1239.20, 1162.39, 1152.92, 1071.37	1203.26 (Cp), 1128.68, 1119.49, 1040.30 (Cp)	1210.05, 1126.69, 1106.82, 1075.05
*v*(C–F)	1297.02	1260.70	1273.37	1306.89	1268.99	1273.15

*ν* = stretching vibration; *b* = bending vibration.

#### C–H vibrations

As previously reported, the C–H stretching band due to the ferrocene group appeared around 3042–3160 cm^-1^ [45]. Based on our recent analysis, the C–H stretching mode ferrocene of **Fc1** is found at 3148.63 cm^-1^ (IR) and at 3149.00 cm^-1^ theoretically. Likewise, the bands observed at 3063.70 cm^-1^ in the IR spectrum and computed at 3063.88 cm^-1^ (DFT) are assigned as the C–H stretching mode within the Cp rings of ferrocene for **Fc2**. Moreover, the weak intensity absorption bands which appears in the range of 2900–3200 cm^-1^ wave number are assigned to the aromatic C–H stretching group [46]. In the present study, the weak band of C–H stretching modes are observed at 3087.05 cm^-1^ and 2839.70 cm^-1^ for **Fc1** and **Fc2**, respectively in the IR spectra, while the calculated bands (DFT) are computed individually at 3087.05 cm^-1^ (**Fc1**) and 2839.66 cm^-1^ (**Fc2**). These correlations assigned values result from the C–H symmetric stretching band located on the fluorophenyl ring for **Fc1** and arise from CH_3_ symmetric stretching band positioned on the methoxy group for **Fc2**. Additionally, the absorption band of in-plane and out-of-plane aromatic C–H bending vibrations are expected to lie down in the region 1000–1300 cm^-1^ and 670–950 cm^-1^, respectively [47, 48]. By referring to **Table 3**, the scissoring and rocking in-plane C–H bending vibrations of **Fc1** are calculated theoretically in the range from 1040–1156 cm^-1^ and 1208–1250 cm^-1^, respectively which can be observed at 1031.42, 1145.86, 1215.98 and 1250.25 in IR spectra. Similarly, the theoretical calculated wave-numbers of *b*(C–H) in-plane for **Fc2** fall from 1040–1120 cm^-1^ (scissoring) and 1128–1203 cm^-1^ (rocking), while the weak intensity bands are observed at 1075.05, 1106.82 and 1210.05 cm^-1^ (IR). However, the medium intensity band at 1126.69 cm^-1^ in the IR spectrum of **Fc2** has been tentatively assigned to the rocking vibration of the methyl in the methoxy group when mixed with the C–O stretching mode.

#### C = O and C–O vibrations

The characteristic band of the carbonyl stretching vibrations for the enones (= C–C = O) substituted chalcones can be found to absorb IR strongly in the region 1600–1750 cm^-1^ [49]. The main common feature of the IR spectra of **Fc1** and **Fc2** is the observed intense peak at 1650.04 and 1651.97 cm^-1^, respectively which are attributed to the saturated carbonyl group. The appearance of strong band is due to the large dipole moment since the carbonyl carbon and oxygen have a large partial positive and negative charge, respectively. Thus, the electronegativity difference between these two atoms is significant for the reason that the wavenumber of the *v*(C = O) owing to the carbonyl group is aforesaid to mainly depends on the bond strength [50]. Comparably, these values are in agreement with the theoretical results, 1666.26 cm^-1^ (**Fc1**) and 1668.65 cm^-1^ (**Fc2**). In consequence of the substitution of methoxy group at the fluorophenyl ring, compound **Fc2** will experience the C–O stretching mode. Generally, the *v*(C–O) can be spotted in the region of 1200–1300 cm^-1^ having a medium to strong peak [51]. In such a way, a medium intensity band is observed experimentally at 1275.15 cm^-1^ in the IR spectrum with the theoretical value at 1268.99 cm^-1^.

#### C = C vibrations

The C = C stretching modes pertaining to the ethylene bridge are expected to be around 1575–1625 cm^-1^ when conjugated with a carbonyl group [52]. The vibrations are highly sensitive to the degree of charge transfer between the donor and acceptor groups which caused the formation of heavy doublet. As predicted from references, the intense band of **Fc1** can be observed at 1593.17 cm^-1^ (IR), whereas the calculated wavenumber is obtained at 1593.95 cm^-1^ (DFT). Likewise, the C = C stretching mode of **Fc2** is characterized by the prominent peak at 1591.33 cm^-1^ in the IR spectrum and calculated theoretically at 1593.34 cm^-1^. Thus, the activation of C12 = C13 stretching mode in IR spectra confirms the charge-transfer interaction between C = O group and fluorophenyl ring *via* the ethylene bridge [53].

### Nuclear Magnetic Resonance (NMR) analysis

The confirmation of hydrogen and carbon framework of **Fc1** and **Fc2** were elucidated by Nuclear Magnetic Resonance (NMR) analysis and presented in **(S2 Fig in S1 File)**. The sets of proton and carbon peaks belonging to the ferrocenyl and the chalcone fragments are identified on the ^1^H NMR and ^13^C NMR spectra, respectively. In addition, the experimental findings also further comparable with DFT calculation performed at B3LYP/6-311G++(d,p). Analysis of ^1^H NMR showed that the ferrocenyl fragment for both compounds are observed at downfield NMR spectra range from 4.2 to 5.0 ppm as singlet peaks, integrating for 9 protons. The corresponding calculated DFT values obtained from range 3.42 to 4.82 ppm (**Fc1**) and 3.39 to 4.82 ppm (**Fc2**). The chemical shifts of the chalcone backbone particularly for H-*α* peak are identified at 7.27 ppm (**Fc1**) and 7.35 ppm (**Fc2**) which both appeared as singlet. Meanwhile, deshielding by the fluoro (R-F) and methoxy (R-OCH_3_) substituents move the NMR signal of proton H-*β* towards a lower field than the H-*α* which can be found at 6.55 ppm (**Fc1**) and 6.53 ppm (**Fc2**) as small doublets in the spectrum. Nevertheless, the calculated NMR values are seen to be increased slightly to downfield at H-*α* 8.14 ppm (**Fc1**), 7.97 ppm (**Fc2**) and H-*β*: 7.33 ppm (**Fc1**), 7.03 ppm (**Fc2**). This downfield chemical shift of H-*α* and H-*β* is caused by the existence of *meta*-protons at the phenyl ring which is influenced by the adjacent R-group, also due to the conjugation of the system [54]. Furthermore, the existence of methyl group (CH_3_) in compound **Fc2** is found at the shielded region of 3.87 ppm, depicting an indistinct peak. The ^13^C NMR analysis demonstrates the carbon-ferrocene signals are observed at region 70 to 81 ppm in both compounds. The typical carbonyl carbon of chalcones, usually appears between δ 186.6 and 196.8 in ^13^C NMR spectrum [55]. For ferrocenyl chalcones of **Fc1** and **Fc2**, the carbonyl carbons are found at downfield spectra of 192 ppm, and 194 ppm at DFT theory. In compound **Fc1**, the α- and β- carbon atoms with respect to the carbonyl group revealed characteristic signals of 132.65 ppm and 138.83 ppm, respectively. Meanwhile, signals rise at 127.18 ppm (C-α) and 153.10 (C-*β*) comes from compound **Fc2**. Theoretically, the corresponding calculated DFT signals of α- and β- carbon are found at region of δ 125.21–147.37ppm (**Fc1**) and δ 123.54–146.55ppm (**Fc2**), respectively. All carbon atoms related to the phenyl ring are found in normal ranges.

### Frontier molecular orbitals (FMO) and UV spectral analysis

**Fig 7:**
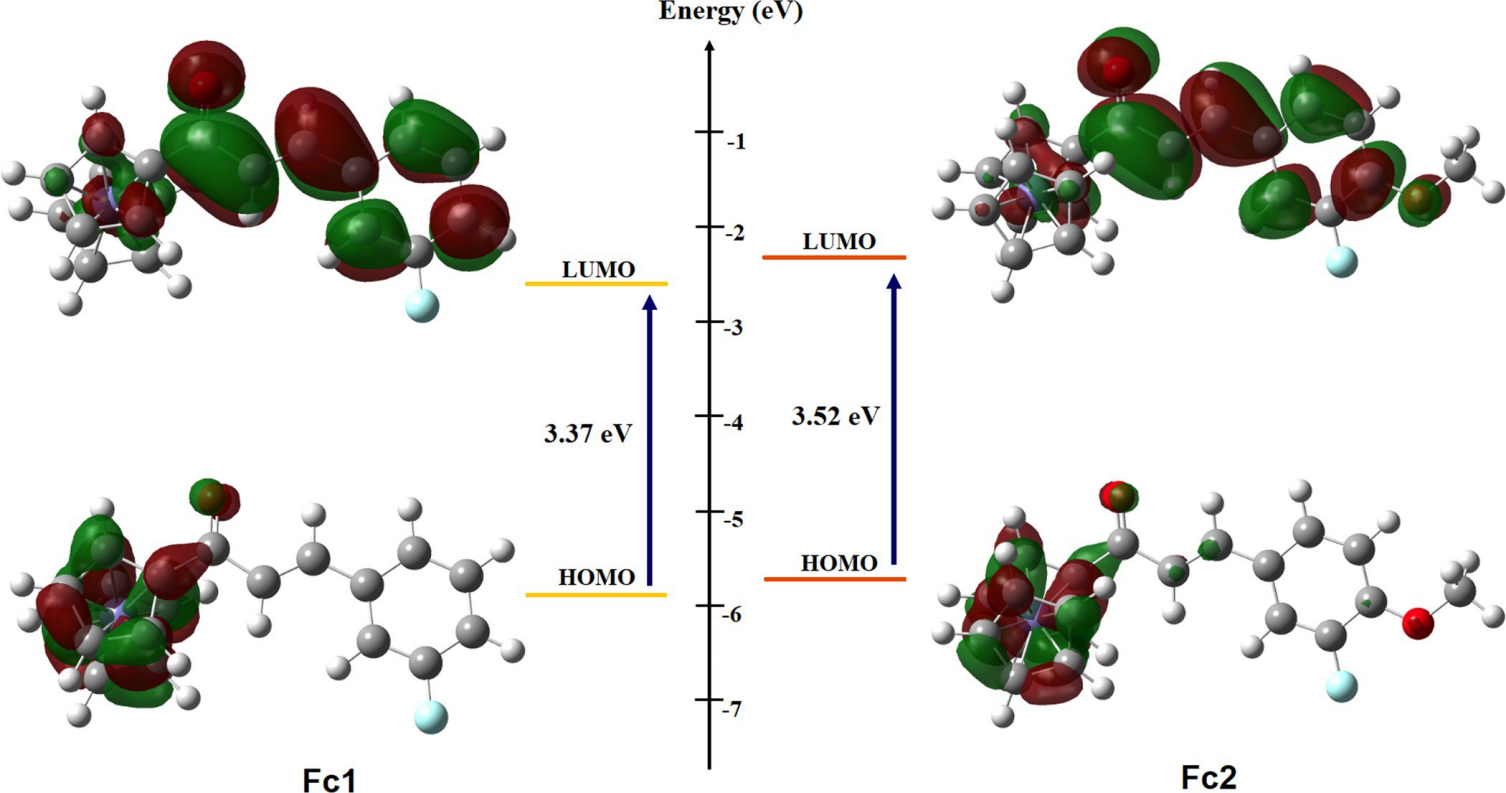
The orbital distribution and energy (in eV) of HOMO and LUMO for Fc1 and Fc2 computed at the B3LYP/6-311G++(d,p) level in gas phase.

The highest occupied molecular orbital (HOMO) and lowest uncopied molecular orbital (LUMO) are the crucial descriptors associated to the reactivity of molecules [56]. The HOMO seems to be one of the important aspects to have the ability as electrons donating in order to empty molecular orbitals with low energy of convenient molecules. Based on **Fig 7**, the distribution of HOMOs and LUMOs for both **Fc1** and **Fc2** present the almost identical to each other. In particular, the HOMO orbitals are mainly accumulated on the donor ferrocene derivatives, whereas the LUMO orbitals are delocalized over the entire molecule with mostly into the acceptor part and enone moiety. In particular, the large parts of the HOMO (-5.825 and -5.755 eV) are delocalized on the ferrocene derivatives, upon removing of an electron from the HOMO in the cationic form leads to elongation of the charge transfer to LUMO level. As for **Fc2**, some electron clouds are accumulated at the oxygen atom of methoxy group, indicating that the existence of the methoxy group apparently imparts the intramolecular charge transfer (ICT) property. An increment of 0.07 eV HOMO level in **Fc2** from **Fc1** supports the above-mentioned statement that the electron-donating group of methoxy will upshift the energy levels with the HOMO level more than the LUMO level [15].

**Fig 8:**
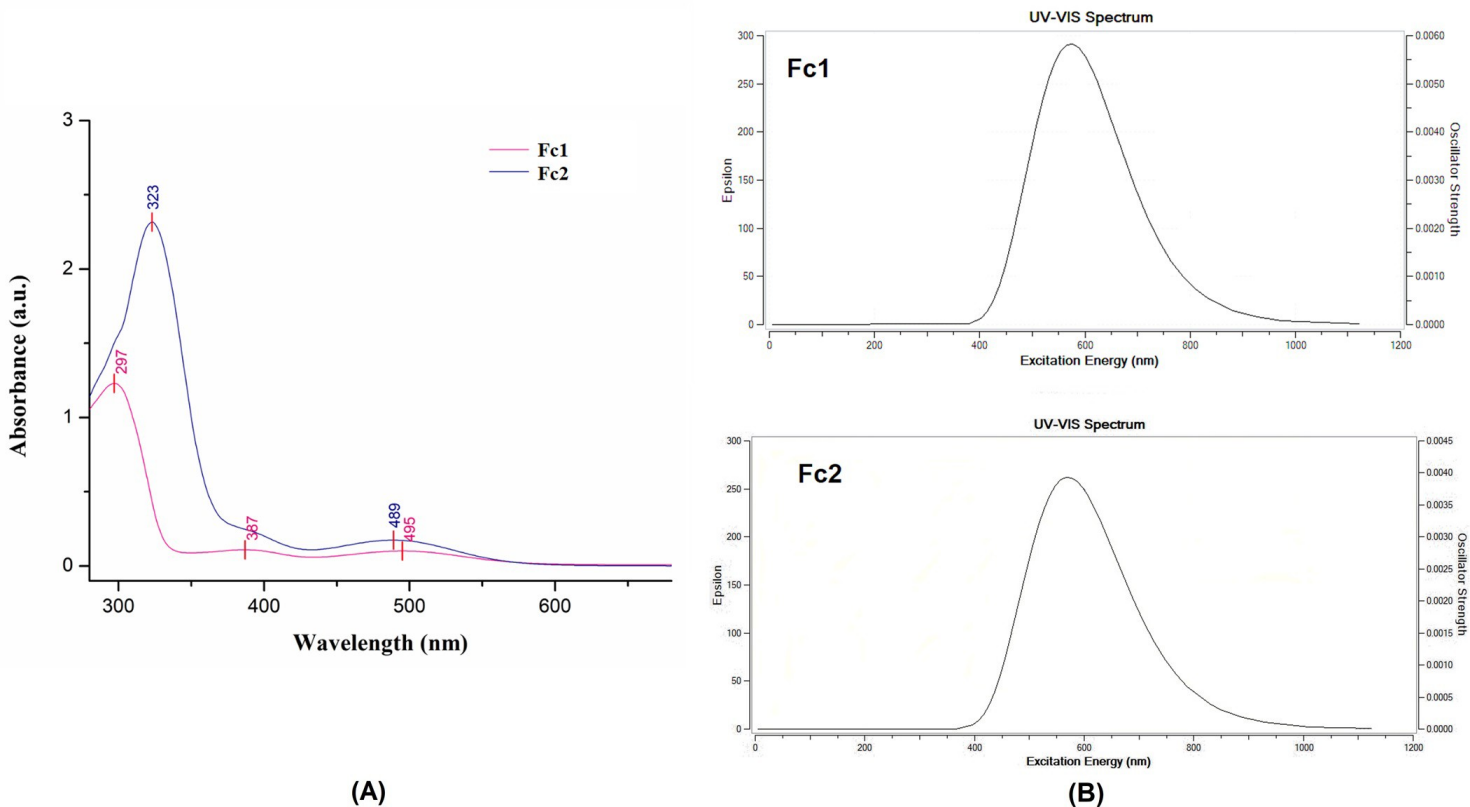
UV-Vis spectra of Fc1 and Fc2. (A) The experimental UV-Vis spectra recorded in acetonitrile. (B) The theoretical UV-Vis spectra using B3LYP/6-311G++(d,p).

In order to ensure consistency also for comparison purposes, the UV-Vis absorption spectra of both synthesized ferrocenyl chalcones **Fc1** and **Fc2** were recorded in acetonitrile. UV-Vis studies revealed two characteristic peaks for **Fc1** at 495 and 297 nm, while **Fc2** at 489 and 323 nm, respectively with an absorption tail which stretches till ±560 nm as shown in **Fig 8**. For compound **Fc1**, the absorption maximum (λ_max_) peak appeared shorter (297 nm) than compound **Fc2** (323 nm) which were attributed to π→π* transition of the α,β-unsubstituted carbonyl conjugated with the ferrocenyl moiety. It was found that the substitution of electron-donating group of methoxy into **Fc2** showed a red shift of 26 nm, that means a significant impact on the maximum absorption value has occurred. A large bathochromic shift (*red shift*) of the π→π* transition may indicate that the interaction of the non-bonding d-orbitals of the metal in the ferrocene derivative with a bulky electron-donating group of the side chain on the Cp-ring [57]. According to ligand field theory, the low-energy peaks of ferrocene are assigned to be d—d transitions on Fe and the stronger absorptions to charge-transfer (CT) states [58]. Normally, the assigned d—d transition of the iron in the ferrocenyl moiety can be observed by the broad low-energy features appear as shoulders at about ±490 nm [59, 60]. In the present study, the absorbance bands of broad peaks were observed at 495 nm (**Fc1**) and 489 nm (**Fc2**) which account for the red colour of the compounds. For the molecules investigated, the computed UV-Vis spectra appeared as an intense electronic transition at 574 nm (**Fc1**) and 566 nm (**Fc2**) in gas phase. The maximum absorption wavelength λ_max_ is related to the electronic transition between frontier molecular orbitals HOMO and LUMO [61]. From the UV-Vis analysis, the observed band gap for **Fc1** is 2.51 eV, whereas **Fc2** represent a slightly higher value that is 2.54 eV. However, the calculated DFT method tends to exaggerate the delocalization of the frontier orbitals HOMO-LUMO caused by the self-interaction error, thus resulting in large difference band gap values between the experimental UV-Vis and HOMO-LUMO [37]. This trend could also be seen from the previous study [62, 63].

### Molecular electrostatic potential

**Fig 9:**
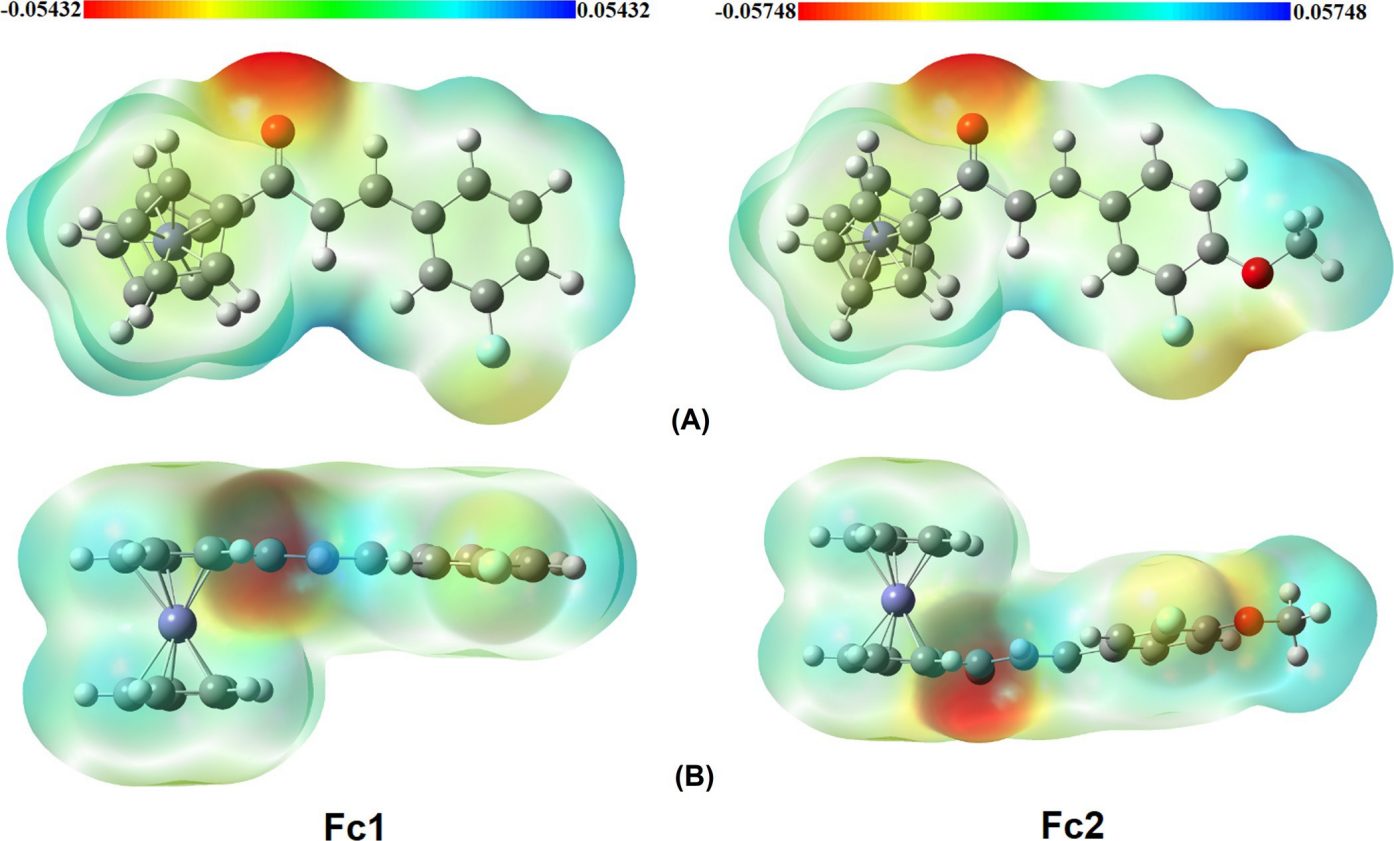
MEP plot of Fc1 and Fc2. (A) From the top view (B) From the side view.

The basic aim for the molecular electrostatic potential (MEP) surface analysis is to locate the positive and negative charged electrostatic potential in the compound by visualizing the colour gradient. The electrostatic potential plot for positive and negative potentials were shown in **Fig 9**. The colour schemes for the MEP surface are red (electron rich or partially negative charge), blue (electron deficient or partially positive charge), light blue (slightly electron-deficient region), yellow (slightly electron-rich region) and green (zero potential region) [64].

The areas of low potential, red are characterized by a relative absence of electrons. The regions are mainly localized over the oxygen atom, O of the carbonyl group with the negative potential values -0.05432 and -0.05748 a.u, which is the site for electrophilic attack for compounds **Fc1** and **Fc2**, respectively (**Fig 9**). The surrounding of the oxygen and fluorine atoms area is reflected as a yellowish colour. The higher electronegativity of -0.05748 a.u for compound **Fc2** is due to the methoxy substituent which contains another one oxygen atom. Additionally, these sites give the information about the region of the molecule that can have the C—H⋯O interactions [65].

In contrast, the positive potential corresponds to the repulsion at the proton by the atomic nuclei (blue region). Furthermore, the positive potential regions are localized over the ferrocene derivatives and phenyl rings which is the possible sites for nucleophilic attack and is reflected as bluish. The maximum positive region of compound **Fc1** and **Fc2** gives the value of 0.05432 and 0.05748 a.u., respectively. The region very near to Fe are positive due to the fact that Fe atom is surrounded by the electropositive hydrogen atoms [66]. However, the carbon and hydrogen benzene having green color are under intermediary potential system.

### Mulliken and ground state dipole moment

**Fig 10:**
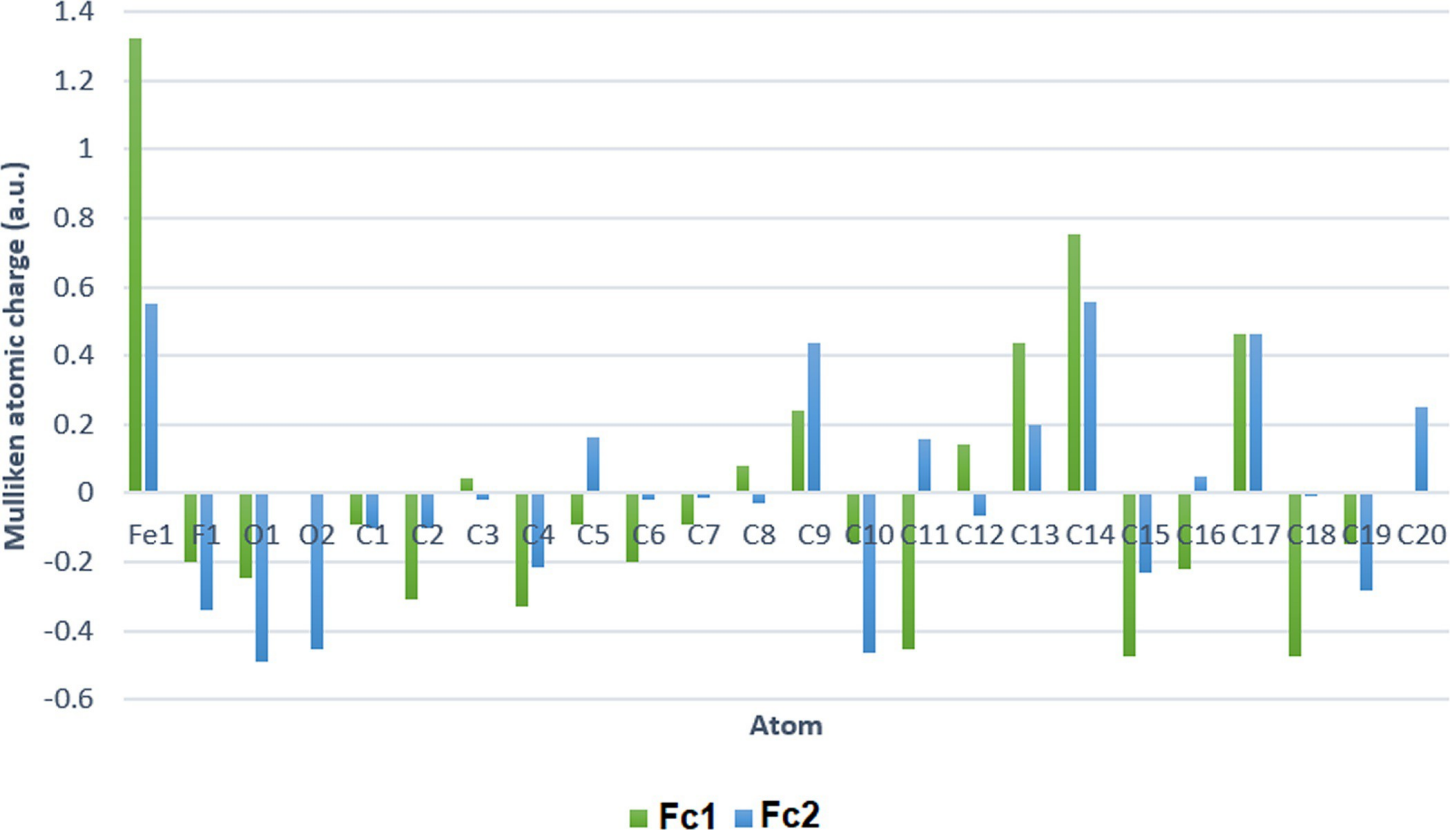
The representation Mulliken population analysis for partial charges of atoms in Fc1 and Fc2.

The natural population analyses of compounds are obtained by Mulliken. It describes the distribution of charges on each individual atom in the molecular orbital. The charge distributions over the atoms in Mulliken calculation are possible to be utilized in estimating the formation of donor and acceptor pairs involving the charge transfer in the molecule. The accumulation of charges on individual atom of **Fc1** and **Fc2** are shown in **Fig 10**and tabulated in the **Table 4**. This Mulliken charge calculation has an important role for the application of quantum chemical calculation of the molecular system [67]. In the calculations of Mulliken charge distributions of the compounds, the red colour indicates for excess of negative charges (-ve) while the green colour indicates for excess of positive charge (+ve) among the bonded atoms. Electrons have the ability to flow from the excess of negative charges’ position to the positive charges’ positions. Commonly, iron presents a positive charge behaving as an electron donor, while cyclopentadienyl Cp represent a negative charge acting as an electron acceptor. It is crucial to be noted, the slightly higher positive charge in Fe atom is observed for **Fc2** (+0.509) compared with **Fc1** (+0.490). As for **Fc1**, the summation of Mulliken charges for carbon atoms between two Cp rings exhibited a huge difference (-1.245, -0.247), contrary with the **Fc2** (-1.219, -0.706). A considerably build-up of negative charge -0.998 on unsubstituted Cp ring of **Fc1** results in lower positive charge at the iron centre, as per above-mentioned. On the other hand, a slight increase of negative charge between the substituted and unsubstituted Cp rings, -0.513 gives rise to higher positive charge of the centred Fe atom in **Fc2**. From Mulliken charge, both compounds exhibited unsymmetrical structure of the ferrocene derivatives. Taking into consideration of Mulliken charge distribution, the substituted Cp ring and 3-fluorophenyl ring in **Fc2** are more conjugated since the dihedral angle between these two planes is almost planar.

**Table 4:** Mulliken’s atomic charges of Fc1 and Fc2 performed at B3LYP methods with 6–311++G(d,p).

Fc1		Fc2	
Atoms	Mulliken atomic charges (a.u.)	Atoms	Mulliken atomic charge (a.u.)
Fe1	1.322	Fe1	0.552
F1	-0.202	F1	-0.341
O1	-0.249	O1	-0.492
C1	-0.092	O2	-0.451
C2	-0.309	C1	-0.103
C3	0.041	C2	-0.103
C4	-0.331	C3	-0.019
C5	-0.093	C4	-0.213
C6	-0.198	C5	0.164
C7	-0.090	C6	-0.018
C8	0.079	C7	-0.011
C9	0.242	C8	-0.029
C10	-0.141	C9	0.436
C11	-0.456	C10	-0.465
C12	0.144	C11	0.159
C13	0.439	C12	-0.067
C14	0.751	C13	0.199
C15	-0.476	C14	0.557
C16	-0.223	C15	-0.230
C17	0.463	C16	0.051
C18	-0.473	C17	0.465
C19	-0.148	C18	-0.004
		C19	-0.285
		C20	0.248

It may be noticed that the all oxygen atoms have negative charge and all carbon atoms have both charges. The results suggested that the oxygen atoms were electron acceptor and charge transfer took place from H to O, which suggests that, the existence of intra and inter-molecular hydrogen bonding in the crystalline phase. Positive charges on C14 atoms for both compounds are greater than those of other atoms because of electron withdrawing groups (oxygen and fluorine atoms) near this atom [68].

**Fig 11:**
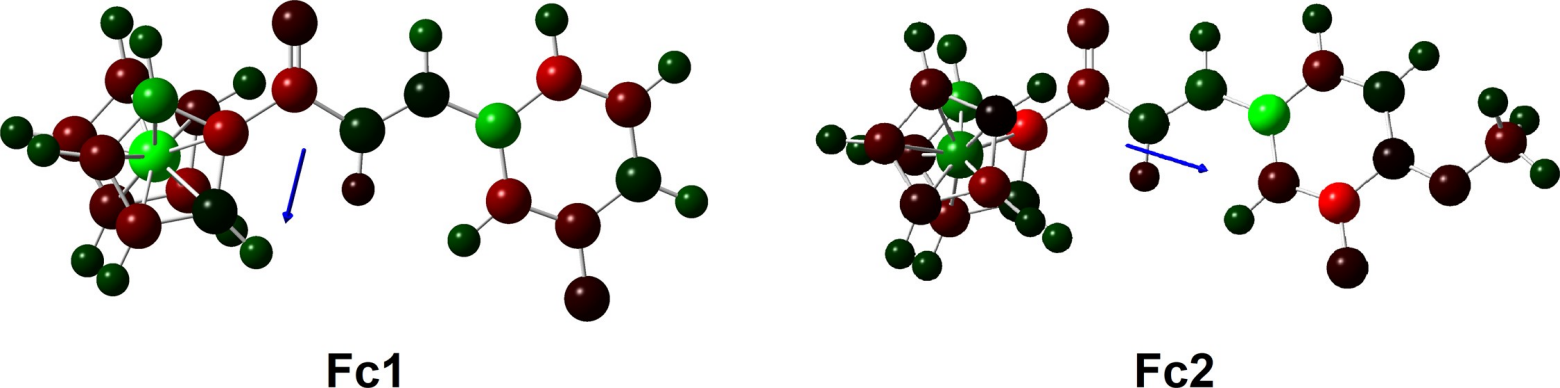
Dipole moment vector for Fc1 and Fc2 at B3LYP/6-311G++(d,p) level of theory.

The total dipole moments and the mulliken structure with different colour of atoms based on the charge have been tabulated in **Table 5**and shown in **Fig 11**, respectively. The dipole vectors are shown as arrows pointing along the bond from the higher electronegative atoms towards the less electronegative atom. Further than that, the length of the arrow is proportional to the magnitude of the electronegativity difference between the atoms in each of molecules. Additionally, the dipole moment increases when a conjugated system is lengthened. Compound **Fc2** (2.35 D) show large dipole moment compared to compound **Fc1** (1.66 D). This is due to the higher total net charge of the dipole moment and the electronegativity difference between atoms in the molecule. The increase of dipole moment in compound **Fc2** is due to the resonance effect contribution of an electron donating methoxy substituent. Furthermore, the–OCH_3_ group is positive with respect to the benzene ring, so that this shift will leave the group more positive leading to an increase in dipole moment [69]. In addition, the dipole moment vector of these molecules are more likely to be in the direction of carbonyl to methoxy.

**Table 5:** The calculated dipole moments (Debye) for all compounds at B3LYP/6-311G++(d,p) level of theory.

Compound	μ_x_	μ_y_	μ_z_	μ_total_
**Fc1**	-0.29	-1.62	0.24	1.66
**Fc2**	-1.86	-1.37	-0.39	2.35

### Photovoltaic performance of DSSCs

**Fig 12:**
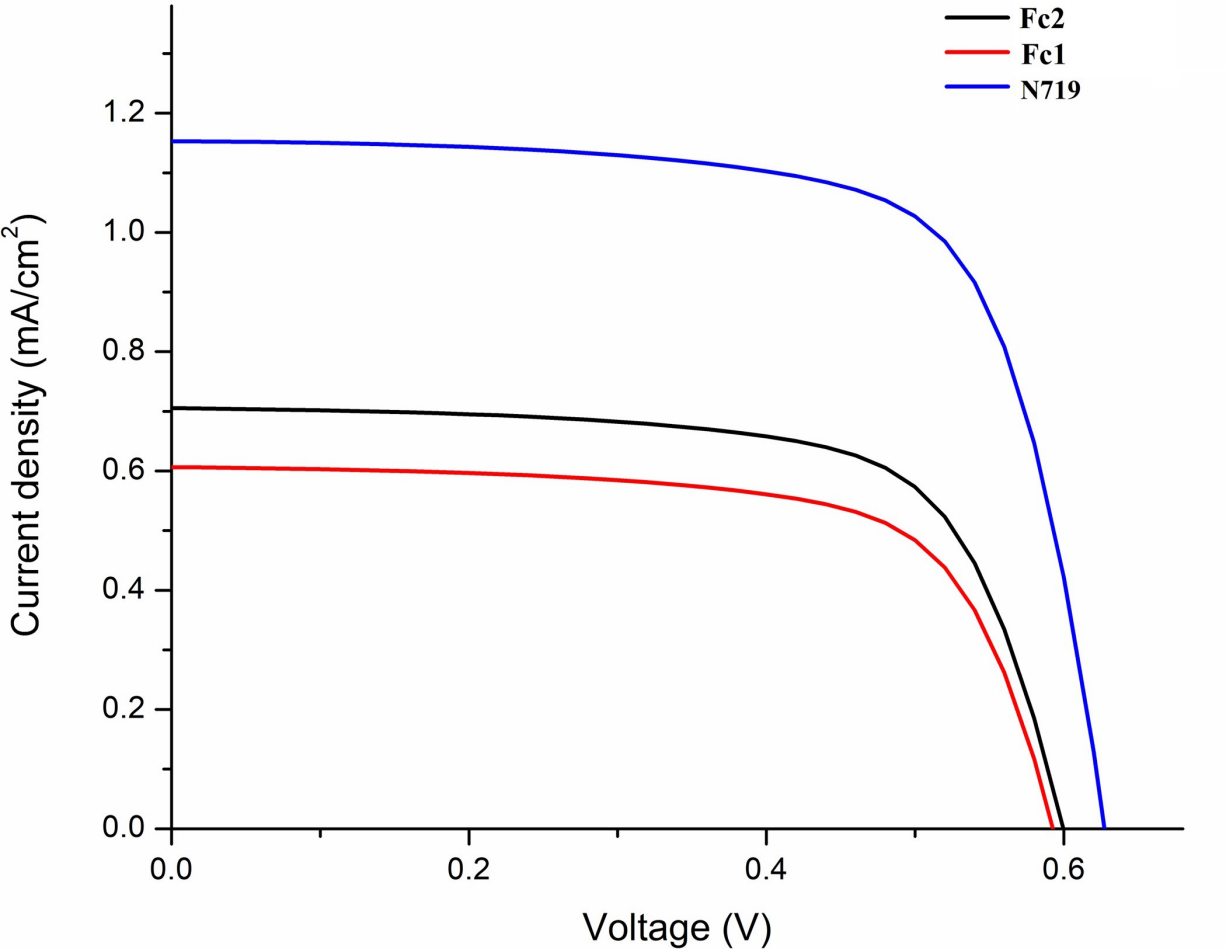
*J*-*V* curves for DSSCs based on Fc1 and Fc2 sensitized photoelectrodes and N719 sensitize photoelectrodes under irradiance.

The photovoltaic effect is the basic of the direct conversion of light into electricity in photovoltaic or solar cells. Each part of solar cell parameters such as the short-circuit current (*I*_sc_), open-circuit voltage (*V*_oc_), fill factor (FF) and efficiency (*n*) is dependent on the solar irradiance level and spectrum of light. The light intensity on a solar cell is associated with the number of suns, in which the standard power density known as AM 1.5 illumination is corresponded to one sun (100 mW cm^-2^). *I*_sc_ vary linearly with light intensity, thus an increase in light illumination level will eventually enhance the current to appear higher. The open voltage (*V*_oc_) is sensitive to the irradiance but not as much as the *I*_sc_. However, the change in temperature that occurred as the irradiance level increases will alter the *V*_oc_ value. **Fig 12**shows the *J*-*V* curves for TiO_2_ –based sandwich-type cells sensitized by two ferrocenyl chalcones under illumination of visible light (λ > 420 nm). The parameters derived from these curves of the DSSC devices are tabulated in **Table 6**. For consistency and comparison, both cells were tested together with the standard dye **N719** under the same conditions. The test for **N719** was conducted thrice with the power conversion efficiency achieved were 0.531, 0.493 and 0.512% for trials 1, 2 and 3, respectively. The small deviation values of three tests denotes that the preparation of DSSC device and physical measurements were consistent.

**Table 6:** Summary of the photovoltaic parameters of the DSSC devices.

Compound	*J*_sc_ (mA cm^-2^)	*V*_oc_ (V)	Fill Factor, FF (%)	Efficiency, *η* (%)
**Fc1**	0.606	0.593	58.70	0.211
**Fc2**	0.776	0.601	52.70	0.246
**N719**	1.153	0.626	70.90	0.512

From the graph plotted, the curves for both ferrocenyl chalcones (**Fc1** and **Fc2**) and **N719** show almost similar trend, in which the increment of *J*_sc_ value gradually shifted *V*_oc_ to the right. The current is higher for the standard dye, **N719** than the ferrocenyl chalcone tested. Nevertheless, compound **Fc2** illustrated a better solar conversion efficiency of 0.246% as compared to **Fc1** (0.211%). This relatively higher photovoltaic performance of **Fc2** may arise due to the planarity of the compound itself. As the evidence suggests that planar compounds promote the ICT to take place efficiently, thus the electronic communication between the ferrocene and substituent phenyl ring is then improved. As previously reported, the presence of the methoxy group (–OCH_3_) to the phenyl ring of the compound was recognized to induce the push-pull effect along the molecule [70]. Consequently, the molecular structure of **Fc1** which is lack from methoxy group anchoring to the fluorophenyl ring results in lower *J*_sc_ and *V*_oc_ rather than **Fc2**. On that account, it impacts the photovoltaic performance of the dye-sensitizer to facilitate the electron transfer towards the semiconductor layer. Nevertheless, under the same conditions, the performances of **Fc1** and **Fc2** are lower than the **N719** dye due to the molecular structure of the **Fc1** and **Fc2** presented herein comprises of fluoro (-F) and fluoro-methoxy (-FOCH_3_) group, respectively but lacks of the -COOH group. The -COOH functional group that exist in the **N719** dye is significance for the rapid electron transport rate. The anchoring group of -COOH is reported to have great potential to combine with the TiO_2_ nanoparticles which can boost the coupling effect of the electrons on the TiO_2_ [71].

Another useful parameter that can be extracted from the IV curve is called the fill factor (FF). FF is essentially an indication on how well the device system work. By considering the FF values of the DSSC devices, dye sensitized of **Fc1** give higher value (58.70%) compared to **Fc2** (52.70%). Initially, the deposition of dye **Fc1** showed deep red colour, whereas dye **Fc2** appeared a slightly pale red colour on the TiO_2_ surface. A good absorption of the dye sensitizer enables more photons to be absorbed, thus enhancing the photocurrent by bringing more electron injection to the nanocrystalline TiO_2_ layer. Due to this reason, compound **Fc2** is capable to yield better overall performance if the dye absorption on TiO_2_ surface is improved.

Validation of the photovoltaic performance was done by comparing all the parameters obtained of the **N719** with the previously reported studies [71, 72]. To analytically solve the single-diode equivalent-circuit model under various illumination, the Shockley **Eq (1)** can be employed for theoretical calculation,

$$V_{\text{OC}}=\frac{kT}{e}\text{ln}\left\{1+\frac{J_{\text{SC}}}{J_{\text{0}}}\left(1-\frac{V_{\text{OC}}}{J_{\text{SC}}R_{\text{P}}A}\right)\right\}=\frac{kT}{e}\text{ln}\;\left(1+\frac{J_{\text{SC}}}{J_{\text{0}}}\right)$$ (1)

Although the photovoltaic testing was conducted under 1 sun of light irradiance, it was noticeable that calculated light intensity obtained only gives value of 40 mW/cm^2^. This low irradiance light might due to the formation of dark current and occurrence of energy degeneracy. Assuming that *J*_SC_ increases linearly with concentration, *J*_o_ does not change with concentration and fill factor remain constant, we can expect the new *V*_oc_ attained are 0.617, 0.639 and 0.650 V for **Fc1**, **Fc2** and **N719**, respectively. As aforementioned above, the *V*_oc_ increases slightly due to temperature change. *J*_SC_ values will be double by 2.5, thus the calculated values achieved are 1.515 mA/cm^2^ (**Fc1**), 1.940 mA/cm^2^ (**Fc2**), 2.88 mA/cm^2^ (**N719**). True to form, all three compounds would experience higher efficiency, 0.549% (**Fc1**), 0.639% (**Fc2**) and 1.33% (**N719**), respectively.

**Fig 13:**
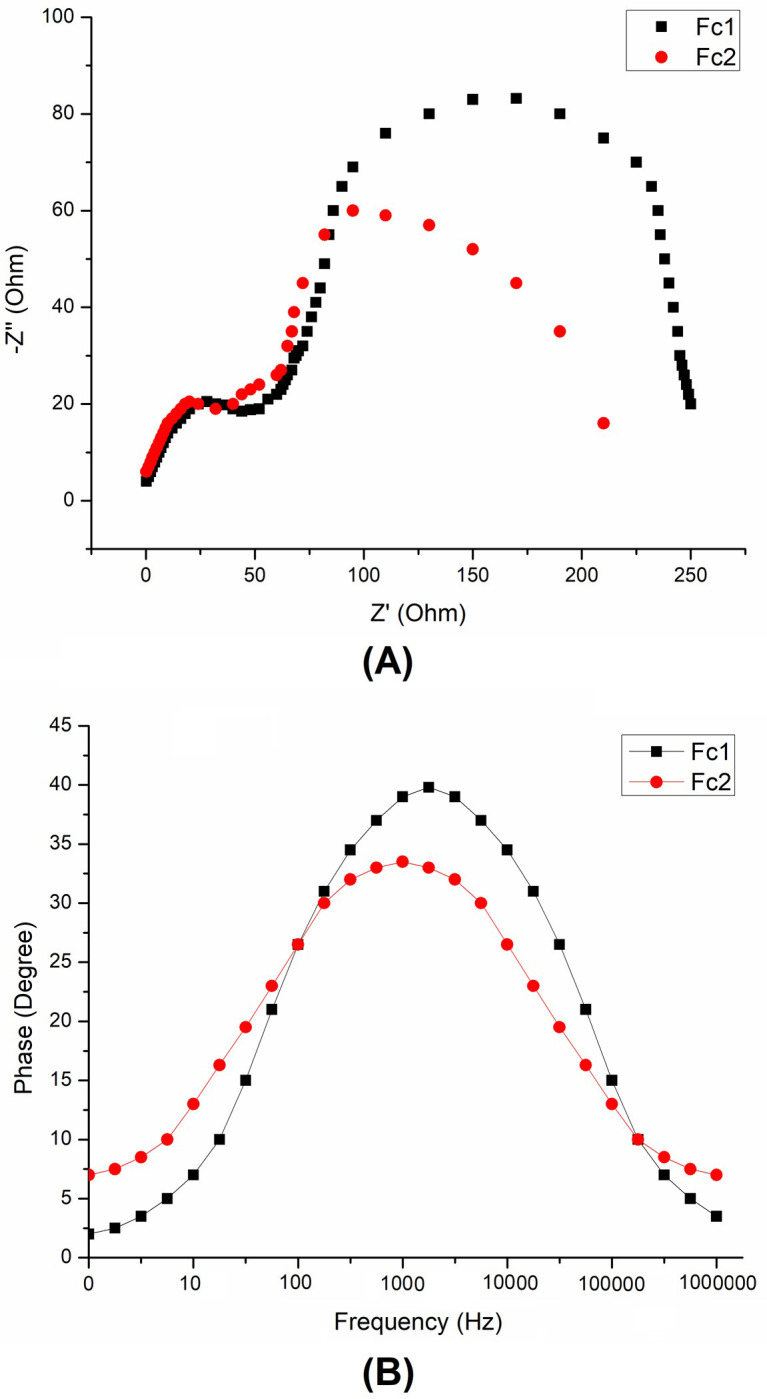
Electrochemical impedance spectra of (A) Nyquist plots (B) Bode-phase plots in DSSCs measured with a 10 mV AC signal under dark conditions.

In DSSCs, the interfacial charge-transfer processes of TiO_2_/dye/electrolyte and its correlation with the cell performance have been investigated by electrochemical impedance spectroscopy (EIS) [71]. The Nyquist plots of EIS for **Fc1** and **Fc2** are shown in **Fig 13A**, measured under dark conditions over a frequency range from 10 mHz to 1 MHz. Two semicircles in the Nyquist plots are observed which correspond to the charge recombination resistance. The small and large semicircles located in the high and middle frequency regions are assigned to the charge transfer at the counter electrode (Pt)/electrolyte and TiO_2_/dye/electrolyte interfaces, respectively. The radius of the semicircle in the middle frequency region is related to the recombination rate in which the larger radius indicates a slower charge recombination [73]. Based on **Fig 13A**, the radius of the large semicircle located in the middle frequency regions in the Nyquist plot for **Fc2** decreases, suggesting a decrease of the electron transfer impedance and an increase of charge transfer rate at this interface.

**Fig 13B** shows the corresponding Bode phase plots which importance to find out the lifetime (τ_e_) of the electrons using the following equation [74].

$${\text{T}}_{e}=\frac{1}{2\pi f_{max}}$$ (2)

Where, *f*_max_ is the frequency at the maximum of the curve in the intermediate frequency region of the Bode plot. The result shows the same style by previously reported study [75] which represents effective suppression of the back-transfer reaction between the ejected electron and the electrolyte.

### Conclusion

Based on the findings in our investigation, it can be concluded that the **Fc2** with methoxy group anchoring to the phenyl ring offers substantial improvements such as the charge transfer between the donor and acceptor, absorption of light in UV-Vis, dipole moment and the photovoltaic performance as compared to the **Fc1**. Despite achieving only ~48% of cell efficiency to **N719** dye, future work is intended to improve so as to consequently increase the photocurrent and power conversion efficiency in DSSCs. From this report, the dihedral angles between enone moiety and the fluorophenyl ring with and without methoxy group substitution have impart the electronic communication and also the charge transfer within the molecules. The planarity of the compound is one of the major components which control the photovoltaic parameters of the DSSC. It is also confirmed that ferrocene derivatives has acted as an excellent donor based from the observed charge accumulation at the LUMO band. Hence, the employment of good acceptor group (-F and -OCH_3_) as substituent atom in **Fc2**, low lying the HOMO-LUMO level (*E*_g_ = 3.52 eV), afford a large dipole moment molecule (*μ* = 2.35) and further extended the visible region for the electronic absorption spectrum (*E*_g_ = 2.54 eV) which propose this ferrocenyl chalcone as an attractive organometallic compound for enhancing the cell efficiency in DSSCs (*η* = 0.246%).

## Supporting information

S1 File (DOCX)
