# Supplementary material for: Comparative analyses of new donor-π-acceptor ferrocenyl-chalcones containing fluoro and methoxy-fluoro acceptor units as synthesized dyes for organic solar cell material
Source: PLoS One. 2020 Nov 4;15(11):e0241113. doi: 10.1371/journal.pone.0241113 (PMC7641456; doi:10.1371/journal.pone.0241113)

**Supporting information for:**

**Comparative Analyses of New Donor-π-Acceptor Ferrocenyl-Chalcones Containing Fluoro and Methoxy-Fluoro Acceptor Units as Synthesized Dyes for Organic Solar Cell Material**

Ainizatul Husna Anizaim^1¶^, Dian Alwani Zainuri^1¶^, Muhamad Fikri Zaini^1¶^, Ibrahim Abdul Razak^1¶^, Hazri Bakhtiar^2,3¶^, Suhana Arshad^1¶^*

^1^ X-ray Crystallography Unit, School of Physics, Universiti Sains Malaysia, USM,

Penang, Malaysia

^2^ Laser Center, Ibnu Sina Institute for Scientific and Industrial Research (ISI-SIR), Universiti Teknologi Malaysia, Johor Bahru, Johor, Malaysia

^3^ Department of Physics, Faculty of Sciences, Universiti Teknologi Malaysia, Johor Bahru, Johor, Malaysia

*****Author to whom correspondence should be addressed; E-Mail: suhanaarshad@usm.my; Tel.: +604-6535652; Fax: +604-6579150.

^¶^These authors contributed equally to this work

**S1 Table. Crystal Refinement Data**

| Refinement Parameters | **Fc1** | **Fc2** |
| --- | --- | --- |
| CCDC deposition numbers | 1945665 | 1911682 |
| Molecular formula | C_19_H_15_FFeO | C_20_H_17_FFeO_2_ |
| Molecular weight | 334.16 | 364.18 |
| Crystal system | Orthorhombic | Monoclinic |
| Space group | *Pna*2_1_ | *P*2_1_/*n* |
| Temperature (K) | 296 | 296 |
| *a*, *b*, *c* (Å) | 20.601 (3), 12.3027 (15), 5.8821 (7) | 11.1429 (8), 7.3917 (5), 20.3228 (15) |
| α, β, γ (°) | 90 | 90, 101.892 (3), 90 |
| *V* (Å^3^) | 1490.8 (3) | 1638.0 (2) |
| *Z* | 4 | 4 |
| Radiation type | Mo *Kα* | Mo *Kα* |
| *µ* (mm^-1^) | 1.02 | 0.94 |
| Crystal size (mm) | 0.56 × 0.13 × 0.04 | 0.51 × 0.28 × 0.06 |
|  |  |  |
| Data collection |  |  |
| Diffractometer | Bruker *APEX*-II CCD | Bruker *APEX*-II CCD |
| Absorption correction | Multi-scan  *SADABS* 2014/5 | Multi-scan  *SADABS* 2014/5 |
| T_min_, T_max_ | 0.691, 0.769 | 0.587, 0.711 |
| No. of measured, independent and  observed [I > 2σ(I)] reflections | 19363, 4324, 2756 | 61658, 4870, 2802 |
| R_int_ | 0.052 | 0.089 |
| (sin *θ*/*λ*)_max_ (Å^−1^) | 0.704 | 0.709 |
|  |  |  |
| Refinement |  |  |
| *R*[*F*2 > 2*σ*(*F*2)], *wR*(*F*2), *S* | 0.051, 0.140, 1.01 | 0.051, 0.159, 1.03 |
| No. of reflections | 4324 | 4870 |
| No. of parameters | 200 | 218 |
| H-atom treatment | H-atom parameters constrained | H-atom parameters constrained |


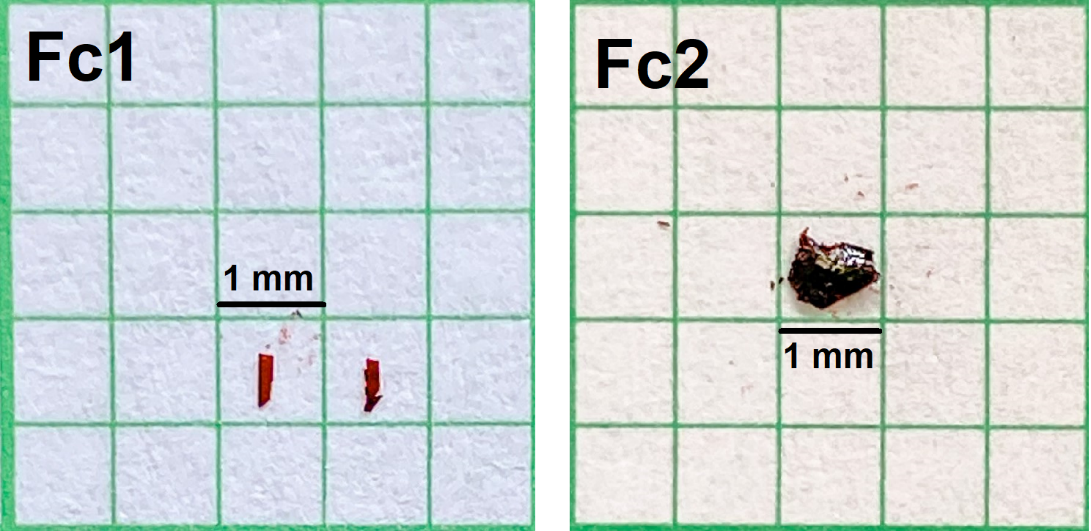


**S1 Fig. The single crystal of Fc1 and Fc2.**


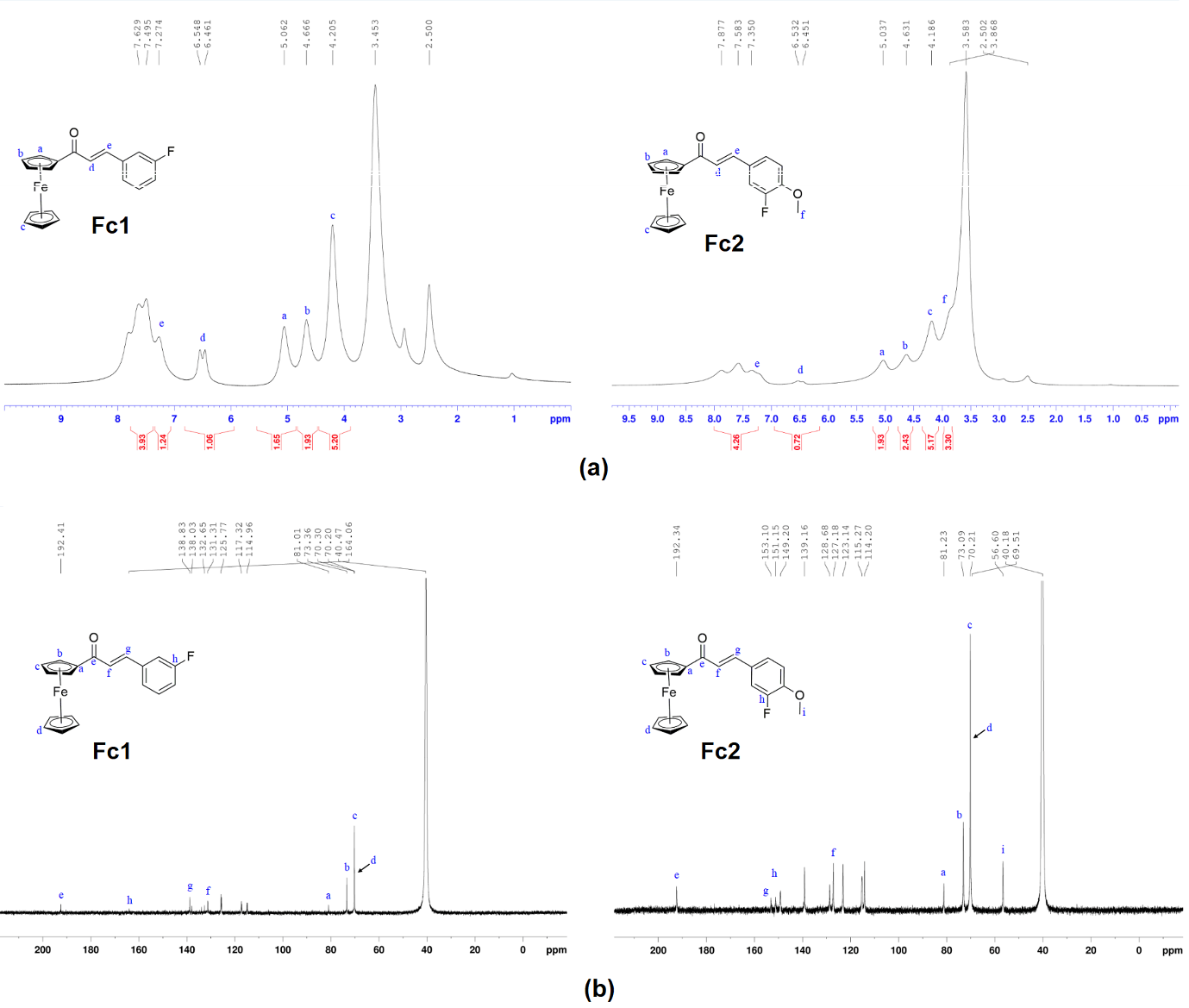


**S2 Fig. NMR Spectra of ferrocenyl chalcone Fc1 and Fc2.** (A) ^1^H NMR (B) ^13^C NMR.

**S1 CheckCIF Validation Reports of Fc1**

# checkCIF/PLATON report

Structure factors have been supplied for datablock(s) mo_fc_test4_0m

THIS REPORT IS FOR GUIDANCE ONLY. IF USED AS PART OF A REVIEW PROCEDURE FOR PUBLICATION, IT SHOULD NOT REPLACE THE EXPERTISE OF AN EXPERIENCED CRYSTALLOGRAPHIC REFEREE.

[No syntax errors found. CIF dictionary](http://www.iucr.org/iucr-top/cif/cif_core/definitions/index.html) [Interpreting this report](http://journals.iucr.org/services/cif/checking/checkcifreport.html)

# Datablock: mo_fc_test4_0m

Bond precision: C-C = 0.0089 A Wavelength=0.71073

| Cell:  Temperature: | a=20.601(3)  alpha=90  296 K | | b=12.3027(15)  beta=90 | | c=5.8821(7)  gamma=90 |
| --- | --- | --- | --- | --- | --- |
|  | Calculated |  |  | Reported |  |
| Volume Space group Hall group  Moiety formula | 1490.8(3)  P n a 21 P 2c -2n C19 H15 F | Fe | O | 1490.8(3)  P n a 21 P 2c -2n C19 H15 F | Fe O |
| Sum formula | C19 H15 F | Fe | O | C19 H15 F | Fe O |
| Mr | 334.16 |  |  | 334.16 |  |
| Dx,g cm-3 | 1.489 |  |  | 1.489 |  |
| Z | 4 |  |  | 4 |  |
| Mu (mm-1) | 1.020 | | 1.020 | | |
| F000 | 688.0 | | 688.0 | | |
| F000’ | 689.65 | |  | | |
| h,k,lmax | 29,17,8 | | 28,17,8 | | |
| Nref | 4352[ 2373] | | 4324 | | |
| Tmin,Tmax | 0.857,0.960 | | 0.691,0.769 | | |
| Tmin’ | 0.567 | |  | | |

Correction method= # Reported T Limits: Tmin=0.691 Tmax=0.769 AbsCorr = MULTI-SCAN

Data completeness= 1.82/0.99 Theta(max)= 30.022 R(reflections)= 0.0513( 2756) wR2(reflections)= 0.1402( 4324)

S = 1.015 Npar= 200

The following ALERTS were generated. Each ALERT has the format

**test-name_ALERT_alert-type_alert-level**.

Click on the hyperlinks for more details of the test.


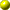
**Alert level C**

[PLAT094_ALERT_2_C](http://journals.iucr.org/services/cif/checking/PLAT094.html) Ratio of Maximum / Minimum Residual Density .... 2.59 Report [PLAT230_ALERT_2_C](http://journals.iucr.org/services/cif/checking/PLAT230.html) Hirshfeld Test Diff for F1 --C18 . 6.7 s.u.

[PLAT241_ALERT_2_C](http://journals.iucr.org/services/cif/checking/PLAT241.html) High ’MainMol’ Ueq as Compared to Neighbors of C1 Check [PLAT242_ALERT_2_C](http://journals.iucr.org/services/cif/checking/PLAT242.html) Low ’MainMol’ Ueq as Compared to Neighbors of Fe1 Check [PLAT334_ALERT_2_C](http://journals.iucr.org/services/cif/checking/PLAT334.html) Small Aver. Benzene C-C Dist C14 -C19 1.37 Ang. [PLAT341_ALERT_3_C](http://journals.iucr.org/services/cif/checking/PLAT341.html) Low Bond Precision on C-C Bonds ............... 0.0089 Ang. [PLAT906_ALERT_3_C](http://journals.iucr.org/services/cif/checking/PLAT906.html) Large K Value in the Analysis of Variance ...... 5.239 Check

**Alert level G**


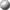
[PLAT794_ALERT_5_G](http://journals.iucr.org/services/cif/checking/PLAT794.html) Tentative Bond Valency for Fe1 (II) . 2.14 Info [PLAT850_ALERT_4_G](http://journals.iucr.org/services/cif/checking/PLAT850.html) Check Flack Parameter Exact Value 0.00 with s.u. 0.05 Check [PLAT883_ALERT_1_G](http://journals.iucr.org/services/cif/checking/PLAT883.html) No Info/Value for _atom_sites_solution_primary . Please Do ! [PLAT910_ALERT_3_G](http://journals.iucr.org/services/cif/checking/PLAT910.html) Missing # of FCF Reflection(s) Below Theta(Min). 1 Note [PLAT912_ALERT_4_G](http://journals.iucr.org/services/cif/checking/PLAT912.html) Missing # of FCF Reflections Above STh/L= 0.600 11 Note [PLAT933_ALERT_2_G](http://journals.iucr.org/services/cif/checking/PLAT933.html) Number of OMIT Records in Embedded .res File ... 1 Note [PLAT978_ALERT_2_G](http://journals.iucr.org/services/cif/checking/PLAT978.html) Number C-C Bonds with Positive Residual Density. 3 Info

0 **ALERT level A** = Most likely a serious problem - resolve or explain

0 **ALERT level B** = A potentially serious problem, consider carefully

7 **ALERT level C** = Check. Ensure it is not caused by an omission or oversight

7 **ALERT level G** = General information/check it is not something unexpected

| 1 | ALERT | type | 1 | CIF construction/syntax error, inconsistent or missing data |
| --- | --- | --- | --- | --- |
| 7 | ALERT | type | 2 | Indicator that the structure model may be wrong or deficient |
| 3 | ALERT | type | 3 | Indicator that the structure quality may be low |
| 2 | ALERT | type | 4 | Improvement, methodology, query or suggestion |
| 1 | ALERT | type | 5 | Informative message, check |

It is advisable to attempt to resolve as many as possible of the alerts in all categories. Often the minor alerts point to easily fixed oversights, errors and omissions in your CIF or refinement strategy, so attention to these fine details can be worthwhile. In order to resolve some of the more serious problems it may be necessary to carry out additional measurements or structure refinements. However, the purpose of your study may justify the reported deviations and the more serious of these should normally be commented upon in the discussion or experimental section of a paper or in the "special_details" fields of the CIF. checkCIF was carefully designed to identify outliers and unusual parameters, but every test has its limitations and alerts that are not important in a particular case may appear. Conversely, the absence of alerts does not guarantee there are no aspects of the results needing attention. It is up to the individual to critically assess their own results and, if necessary, seek expert advice.

## Publication of your CIF in IUCr journals

A basic structural check has been run on your CIF. These basic checks will be run on all CIFs submitted for publication in IUCr journals (*Acta Crystallographica*, *Journal of Applied Crystallography*, *Journal of Synchrotron Radiation*); however, if you intend to submit to *Acta Crystallographica Section C* or *E* or *IUCrData*[, you should make sure that full publication checks](http://journals.iucr.org/services/cif/checking/checkform.html) are run on the final version of your CIF prior to submission.

## Publication of your CIF in other journals

Please refer to the *Notes for Authors* of the relevant journal for any special instructions relating to CIF submission.

## PLATON version of 10/08/2020; check.def file version of 06/08/2020

**Datablock mo_fc_test4_0m** - ellipsoid plot


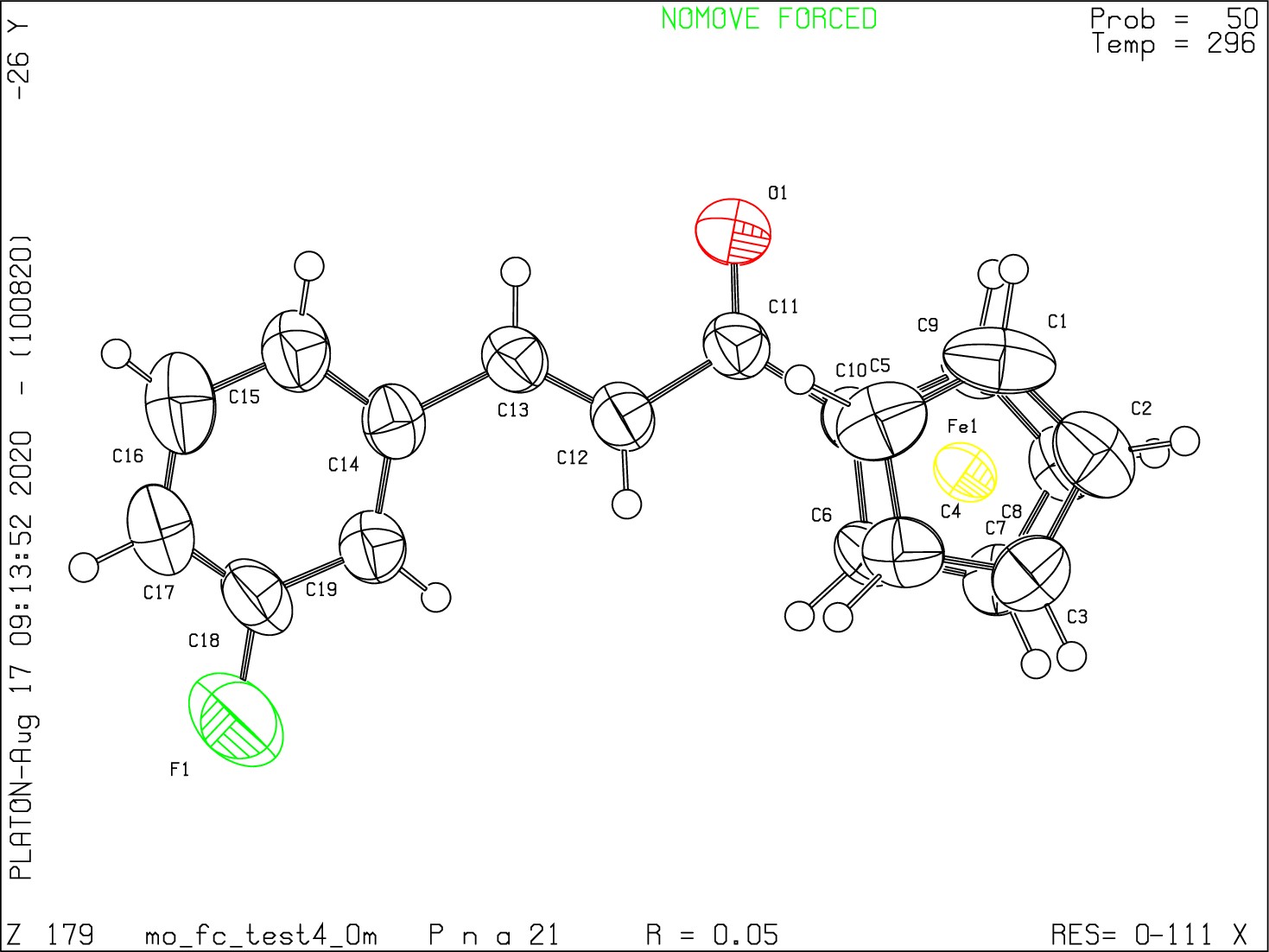


**S1 CheckCIF Validation Reports of Fc2**

# checkCIF/PLATON report

Structure factors have been supplied for datablock(s) mo_AHA8_0m

THIS REPORT IS FOR GUIDANCE ONLY. IF USED AS PART OF A REVIEW PROCEDURE FOR PUBLICATION, IT SHOULD NOT REPLACE THE EXPERTISE OF AN EXPERIENCED CRYSTALLOGRAPHIC REFEREE.

[No syntax errors found. CIF dictionary](http://www.iucr.org/iucr-top/cif/cif_core/definitions/index.html) [Interpreting this report](http://journals.iucr.org/services/cif/checking/checkcifreport.html)

# Datablock: mo_AHA8_0m

Bond precision: C-C = 0.0058 A Wavelength=0.71073

Cell: a=11.1429(8) b=7.3917(5) c=20.3228(15)

alpha=90 beta=101.892(3) gamma=90 Temperature: 296 K

Calculated Reported

Volume 1638.0(2) 1638.0(2)

Space group P 21/n P 21/n

Hall group -P 2yn -P 2yn

Moiety formula C20 H17 F Fe O2 ?

Sum formula C20 H17 F Fe O2 C20 H17 F Fe O2 Mr 364.19 364.18

Dx,g cm-3 1.477 1.477

Z 4 4

Mu (mm-1) 0.939 0.939

F000 752.0 752.0

F000’ 753.70

h,k,lmax 15,10,28 15,10,28

Nref 4891 4870

Tmin,Tmax 0.739,0.948 0.587,0.711

Tmin’ 0.613

Correction method= # Reported T Limits: Tmin=0.587 Tmax=0.711 AbsCorr = MULTI-SCAN

Data completeness= 0.996 Theta(max)= 30.249

R(reflections)= 0.0510( 2802) wR2(reflections)= 0.1594( 4870)

S = 1.029 Npar= 218

The following ALERTS were generated. Each ALERT has the format

**test-name_ALERT_alert-type_alert-level**.

Click on the hyperlinks for more details of the test.


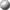


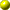

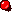
 **Alert level A**

| [PLAT937_ALERT_4_A](http://journals.iucr.org/services/cif/checking/PLAT937.html) | Weight Expression Contains Exponential Term .... | -999.000 | Report |
| --- | --- | --- | --- |
| 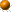 **Alert level**  [PLAT241_ALERT_2_B](http://journals.iucr.org/services/cif/checking/PLAT241.html) | **B**  High ’MainMol’ Ueq as Compared to Neighbors of | C2 | Check |
| [PLAT242_ALERT_2_B](http://journals.iucr.org/services/cif/checking/PLAT242.html) | Low ’MainMol’ Ueq as Compared to Neighbors of | Fe1 | Check |
| **Alert level**  [PLAT213_ALERT_2_C](http://journals.iucr.org/services/cif/checking/PLAT213.html) | **C**  Atom C2 has ADP max/min Ratio ..... | 3.2 | prolat |
| [PLAT220_ALERT_2_C](http://journals.iucr.org/services/cif/checking/PLAT220.html) | NonSolvent Resd 1 C Ueq(max)/Ueq(min) Range | 3.2 | Ratio |
| [PLAT241_ALERT_2_C](http://journals.iucr.org/services/cif/checking/PLAT241.html) | High ’MainMol’ Ueq as Compared to Neighbors of | C1 | Check |
| [PLAT241_ALERT_2_C](http://journals.iucr.org/services/cif/checking/PLAT241.html) | High ’MainMol’ Ueq as Compared to Neighbors of | C8 | Check |
| [PLAT906_ALERT_3_C](http://journals.iucr.org/services/cif/checking/PLAT906.html) | Large K Value in the Analysis of Variance ...... | 2.572 | Check |
| **Alert level**  [PLAT012_ALERT_1_G](http://journals.iucr.org/services/cif/checking/PLAT012.html) | **G**  N.O.K. _shelx_res_checksum Found in CIF ...... | Please | Check |
| [PLAT013_ALERT_1_G](http://journals.iucr.org/services/cif/checking/PLAT013.html) | N.O.K. _shelx_hkl_checksum Found in CIF ...... | Please | Check |
| [PLAT794_ALERT_5_G](http://journals.iucr.org/services/cif/checking/PLAT794.html) | Tentative Bond Valency for Fe1 (II) . | 2.26 | Info |
| [PLAT883_ALERT_1_G](http://journals.iucr.org/services/cif/checking/PLAT883.html) | No Info/Value for _atom_sites_solution_primary . | Please | Do ! |
| [PLAT912_ALERT_4_G](http://journals.iucr.org/services/cif/checking/PLAT912.html) | Missing # of FCF Reflections Above STh/L= 0.600 | 22 | Note |
| [PLAT978_ALERT_2_G](http://journals.iucr.org/services/cif/checking/PLAT978.html) | Number C-C Bonds with Positive Residual Density. | 6 | Info |

1. **ALERT level A** = Most likely a serious problem - resolve or explain
2. **ALERT level B** = A potentially serious problem, consider carefully
3. **ALERT level C** = Check. Ensure it is not caused by an omission or oversight
4. **ALERT level G** = General information/check it is not something unexpected

| 3 | ALERT | type | 1 | CIF construction/syntax error, inconsistent or missing data |
| --- | --- | --- | --- | --- |
| 7 | ALERT | type | 2 | Indicator that the structure model may be wrong or deficient |
| 1 | ALERT | type | 3 | Indicator that the structure quality may be low |
| 2 | ALERT | type | 4 | Improvement, methodology, query or suggestion |
| 1 | ALERT | type | 5 | Informative message, check |

It is advisable to attempt to resolve as many as possible of the alerts in all categories. Often the minor alerts point to easily fixed oversights, errors and omissions in your CIF or refinement strategy, so attention to these fine details can be worthwhile. In order to resolve some of the more serious problems it may be necessary to carry out additional measurements or structure refinements. However, the purpose of your study may justify the reported deviations and the more serious of these should normally be commented upon in the discussion or experimental section of a paper or in the "special_details" fields of the CIF. checkCIF was carefully designed to identify outliers and unusual parameters, but every test has its limitations and alerts that are not important in a particular case may appear. Conversely, the absence of alerts does not guarantee there are no aspects of the results needing attention. It is up to the individual to critically assess their own results and, if necessary, seek expert advice.

## Publication of your CIF in IUCr journals

A basic structural check has been run on your CIF. These basic checks will be run on all CIFs submitted for publication in IUCr journals (*Acta Crystallographica*, *Journal of Applied Crystallography*, *Journal of Synchrotron Radiation*); however, if you intend to submit to *Acta Crystallographica Section C* or *E* or *IUCrData*[, you should make sure that full publication checks](http://journals.iucr.org/services/cif/checking/checkform.html) are run on the final version of your CIF prior to submission.

## Publication of your CIF in other journals

Please refer to the *Notes for Authors* of the relevant journal for any special instructions relating to CIF submission.

## PLATON version of 10/08/2020; check.def file version of 06/08/2020

**Datablock mo_AHA8_0m** - ellipsoid plot


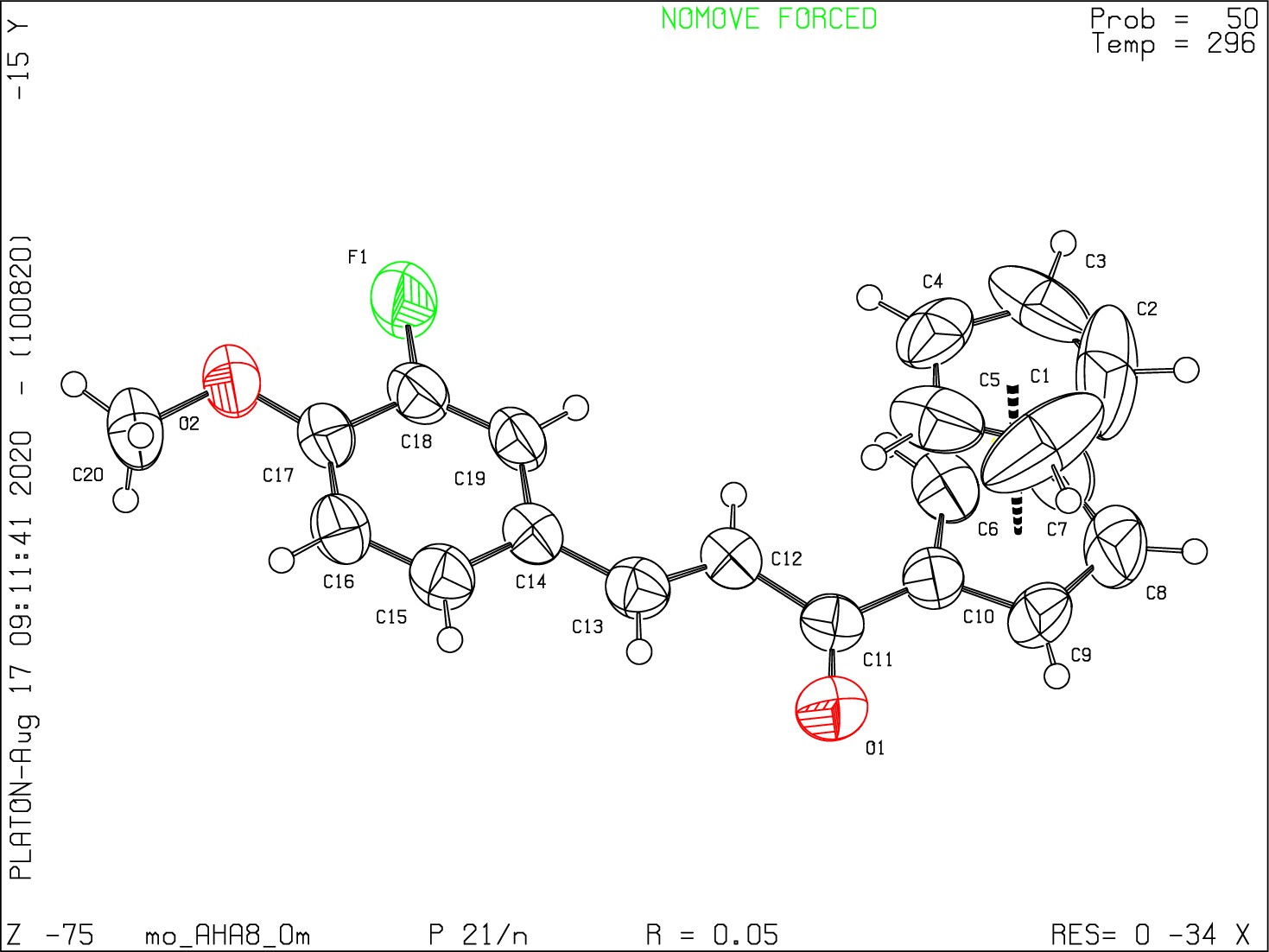

Supplement: S1 File — (DOCX) [file pone.0241113.s001.docx]
